# Multiport bidirectional converters for off board charging stations of electric vehicles

**DOI:** 10.1038/s41598-025-21893-8

**Published:** 2025-10-16

**Authors:** Hazem H. Mostafa, Amr M. Ibrahim, Fathy Z. Amer, Eman F. Sawires

**Affiliations:** 1 Energy and Renewable Energy Department, Faculty of Engineering, Egyptian Chinese University, Cairo, Egypt; 2 Electrical Power and Machines Department, Faculty of Engineering, Ain Shams University, Cairo, Egypt; 3 The Electronics and Communications Engineering Department, Faculty of Engineering, Helwan University, Cairo, Egypt

**Keywords:** Bi-directional dc converter, Multi-port, PV2V, G2V, V2G, PV2G, Electrical and electronic engineering, Solar cells

## Abstract

In this paper, two multi-port bi-directional converters are proposed to be utilized as off-board Electric Vehicles (EVs) charging station. Both converters are designed to integrate renewable energy along with grid power. High gain, three ports, and four modes of operation are the main advantages of these converters. The first mode is Photovoltaic-to-Vehicle (PV2V), the second is Grid-to-Vehicle (G2V). In addition, both converters support Vehicle-to-Grid (V2G) mode (third mode) among with PV-to-Grid (PV2G) mode which is the fourth mode. Theoretical analysis and simulation are done in continuous current mode. Theoretical analysis and simulations show that both converters have the same gain in all four modes, however Boost-Boost-Buckboost (BBB) converter has higher efficiency and lower number of components than that of the Boost-Boost-SEPIC (BBS) converter. Theoretical Border Conduction Mode (BCM) and Discontinues Conduction Mode (DCM) is also conducted for BBB-MPB. A prototype is implemented for the BBB converter. The prototype is tested for all operating modes as the operating voltage is 56.5 V, the output voltage is measured to be 17.7 V in G2V, and PV2V, 158.3 V in V2G, and for the last mode PV2G the output is 102.83 V. The maximum efficiency is calculated to be 94.3%. Compared to latest converters, the proposed BBB converter has higher number of ports, higher number of operating modes and also higher efficiency. Small signal analysis and dynamic load response is studied for the proposed converter and show that the converter has a marginal stability. According to this analysis the converter needs a control algorithm to overcome the marginal stability.

## Introduction

The escalating impact of greenhouse gases emitted by traditional internal combustion engines raises environmental concerns. This led to the rise of environmentally friendly Electric Vehicles (EVs) in the automotive sector^1–3^. Nevertheless, charging EVs batteries via the utility grid increases the demand on the grid and ultimately raises the electricity costs for EV owners, hence requiring the utilization of other energy sources^4,5^. Renewable Energy Sources (RESs) are both unlimited and pollution-free, making them suitable for charging EV batteries.

EVs are equipped with on-board systems. The primary benefits of this category are the capability to immediately connect to the grid without the requirement of constructing a charging station. Nevertheless, the drawbacks of this charging technology include the requirement to install an on-board charger in each EV, resulting in higher production costs, as well as the limited power and length of battery charging.

EV charging in off-board systems is performed via dedicated charging stations. For off-board charging, the charging station include all the equipment involved in charging EVs. Consequently, the production cost of EVs is lowered in comparison to conventional ones. In addition, the efficiency and power density are enhanced in comparison to on-board systems, leading to a significant reduction in charging time^6–9^.

The integration of solar energy with EVs charging is essential for diminishing our dependence on fossil fuels. Electrical power is derived from several sources, and it is crucial that the EV is charged by sustainable energy. With the increasing popularity of EVs, it is anticipated that nearly every individual who possesses a solar panel will have a solar charging station installed in their residence within the next several years. Smart grid-connected PV arrays provide efficient EV charging by synchronizing with daily energy demand patterns. A surplus of PV power during periods of high solar activity is smoothly integrated into the utility grid, allowing for net metering advantages even while cars are being used. Once back home, the collected credit counterbalances the electric vehicle charging by facilitating bidirectional power transfer, so efficiently utilizing home-generated solar energy for EV mobility^10–14^.

Moreover, bidirectional charging facilitates power transmission in both directions, enabling the EV to not only receive power but also provide power to the grid or other devices. This topology is advantageous for Vehicle-to-Grid (V2G) integration, wherein EV can function as energy storage units and supply electricity to the grid during periods of high demand or assist in meeting local energy requirements. Bidirectional charging necessitates the inclusion of supplementary hardware and control systems in the charging infrastructure^15–20^.

A different methodology is suggested as a replacement for the development of a power converter with numerous integrated ports, namely Multi Port (MP) converters. The aim of the MP converter is to create linkages between several power sources and input/output devices by usage of a single power converter. This facilitates the transmission of electrical energy between every individual port. The objective of these MP converters is to remove superfluous semiconductor switching devices often employed in conventional topologies and superfluous power conversion stages^21–25^.

The integration of Bi-Directional Converter (BDC) with the MP converter is introduced. A MP converter with three-ports for EV charging from PV panels is proposed in literature. This MP converter considers various technical aspects to determine the efficiency and enhance the design. In addition, a BD multilevel inverter with fewer switches is proposed. This multi-level inverter is suitable for medium voltage applications. MP converters with bi-directional capabilities offer several advantages compared to traditional ones. These advantages include the ability to integrate renewable energy sources, grid, and loads, resulting in improved system efficiency, reduced complexity and costs, and improved power management. These benefits are crucial for the development of energy systems and the integration of renewable energy^26,27^.

To address the key limitations of existing converters such as high number of component, low efficiency (below 94.5%)^19–26^, excessive voltage/current stresses, limited input/output ports (limited to 2 ports)^19,20^, and restricted operational modes (only 2 modes)^19–27^, this paper introduces two novel non-isolated Multiport Bidirectional (MPB) converters. These converters are uniquely designed to enhance the integration of Renewable Energy Sources (RES) with EV charging stations. Unlike conventional isolated topologies, the proposed non-isolated MPB converters achieve a remarkable peak efficiency of 94.3% surpassing existing solutions in literature while maintaining significantly lower current/voltage ripples.

Their compact, transformer-less design reduces cost, improves power density, and simplifies control without compromising performance. The converters support bidirectional power flow with versatile step-up/step-down operation and four distinct modes: Photovoltaic-to-Vehicle (PV2V), Grid-to-Vehicle (G2V), Vehicle-to-Grid (V2G), and Photovoltaic-to-Grid (PV2G).

Compared to prior MPB converters, the proposed topologies offer superior voltage gain, higher efficiency (94.3%), reduced ripples, and fewer components leveraging the inherent advantages of non-isolated structures (lower magnetic losses, higher reliability). This work bridges a critical gap in RES-EV systems by delivering a high-performance, cost-effective, and adaptable power conversion solution. The proposed converter can offer an all-in-one solution for off-board EV charging stations.

The proposed converter enables integrated PV-battery-grid operation and dynamic 200–1000 V output for legacy/800V EVs without extra stages, surpassing off-the-shelf chargers requiring external buffers and hardware swaps. Its renewable-optimized modes (PV2V/G2V) further streamline solar-powered charging. However, it’s discrete design currently trails commercial SiC/GaN modules by 2–3% efficiency and carries higher costs at scale^28^.

To ensure robust performance, a small-signal analysis is conducted, evaluating the converter’s dynamic response under varying load conditions. The study illustrates that the proposed converters have a marginal stability. As the stability is marginal or low, the converter needs a control algorithm to overcome this stability issue^29,30^. Future work aims to enhance the system’s performance by combining several optimum control algorithms. This integration will enable real-time, dynamic management and smooth transitions between different modes. Additionally, an MPPT algorithm will be included to optimize the power output of the PV source. While no soft-switching techniques are included in this study, the topology can be further enhanced in future work by integrating soft switching schemes to reduce losses and improve efficiency.

## The proposed converters topology analysis

### Boost-boost-buckboost

**Fig. 1:**
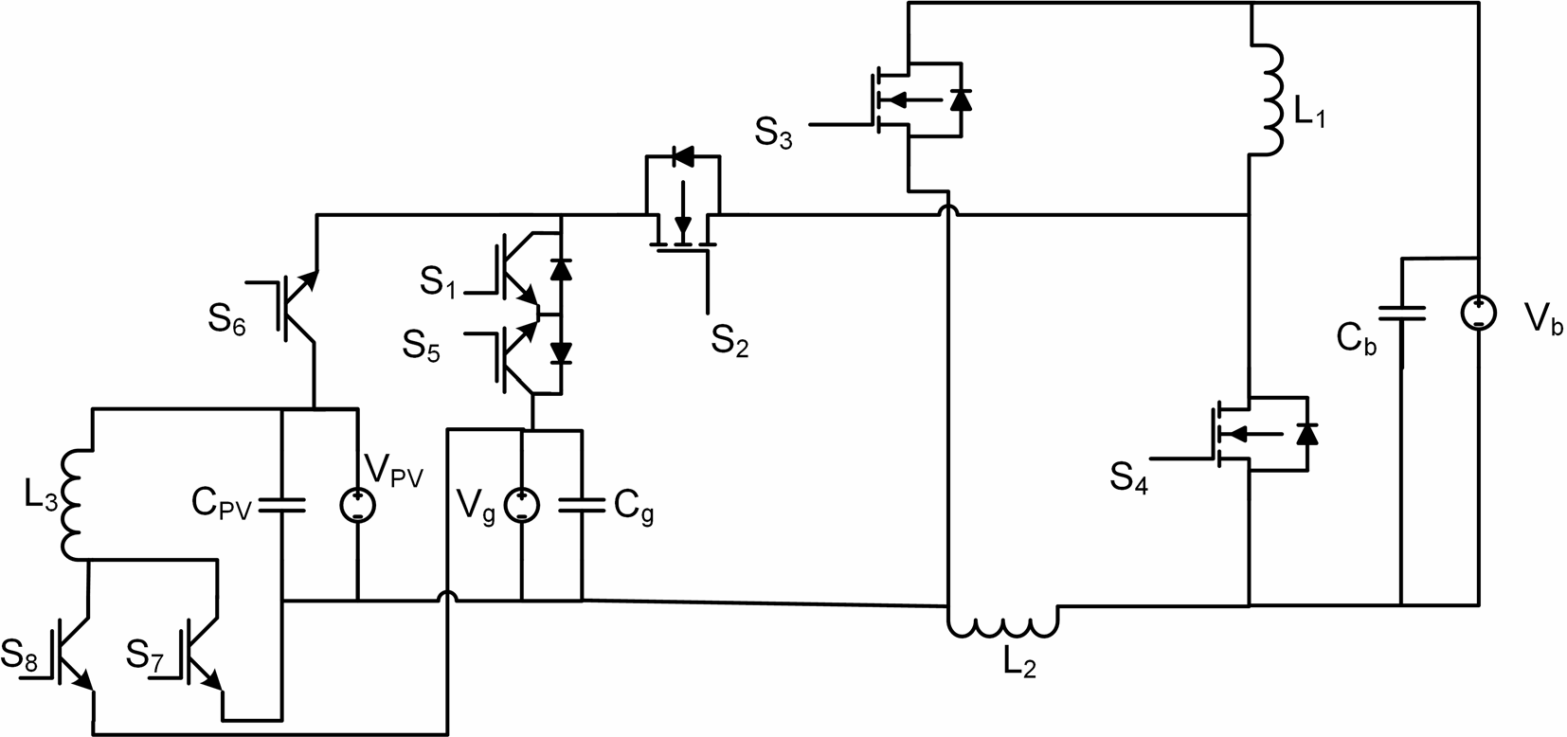
Boost-Boost-Buckboost converter circuit.

Based on a combination between boost and buck boost, the proposed Boost-Boost-Buckboost (BBB) MPB is configured. This combination enables the achievement of a high voltage gain. The proposed converter consists of three MOSFETs (S_2_, S_3_, and S_4_), five IGBTs (S_1,_ S_5,_ S_6,_ S_7_ and S_8_), three identical inductors (L_1_, L_2,_ L_3_), and three capacitors are used as a filter (C_pv_, C_g_, C_b_), as shown in Fig. 1. In each mode, two time intervals are identified. The first time interval (t_0_, t_1_) is nominated as T_i1_ and the second time interval (t_1_, t_2_) is nominated as T_i2_. In this paper, the Continues Conduction Mode (CCM) is investigated due to the nature of the MPBs, which is used to charge a battery or supply power to the grid from either the PV or the battery. In addition, Discontinues Conduction Mode (DCM) and Boundary Conduction Mode (BCM) have higher peak currents, higher output voltage ripples and higher input current ripples.

S_6,_ S_7_ and S_8_ are chosen to be IGBTs. In fact that IGBTs naturally block reverse current, eliminating the need for additional components. In mode 1, power flows from PV2V. In mode 4, power flows from PV2G. These two modes have a unidirectional power flow. The intrinsic body diode in power MOSFETs is incompatible with the unidirectional switching requirement for some modes of the proposed BBB converter. Therefore, IGBTs, which lack this feature, are employed for critical modes such as PV2G and PV2V to enforce strict unilateral current flow. In contrast, BJTs are current-controlled devices suffering from higher base drive losses and slower switching speeds, making them inefficient for high-frequency DC-DC conversion.

#### First and second modes (step-down) of operation

**Fig. 2:**
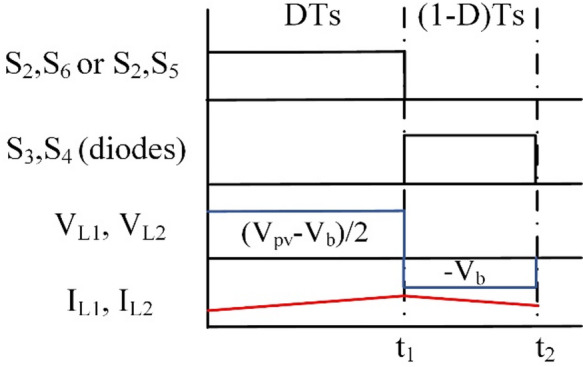
Wave forms of BBB operation in modes 1 and 2.

In step-down operation, the waveforms can be inferred as presented in Fig. 2.

**Fig. 3:**
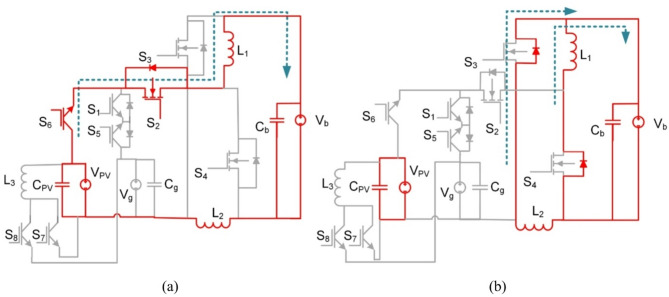
BBB converter operation in modes 1 and 2 (**a**) Interval T_i1_, (**b**) Interval T_i2_.

At T_i1_: S_2_, and S_6_ for the PV2V mode (S_2_, and S_5_ for the G2V mode) are on, and the rest of the switches are off. The First source (V_pv_ or V_g_) charges L_1_ and L_2_. The circuit of BBB and its operation are shown in Fig. 3(a). The following Eq. (1) is the expression for the voltages of the inductors L_1_ and L_2_.

1 $$\:{V}_{L1}={V}_{L2}=\frac{{V}_{pv}-{V}_{b}}{2}$$

As for T_i2_: current flows through S_3_ and S_4_ diodes to V_b_ while the rest of the switches are off. The battery is supplied by a discharge current from L_1_, L_2_ as shown in Fig. 3(b). Voltages of inductors L_1_ and L_2_ are expressed as follows:

2 $$\:{V}_{L1}={V}_{L2}=-{V}_{b}$$

The gain of BBB converter in modes 1 and 2 (step-down) are obtained as follows:

3 $$\:\frac{{V}_{b}}{{V}_{pv}}=\frac{D}{2-D}$$

#### Third mode (step-up) of operation

**Fig. 4:**
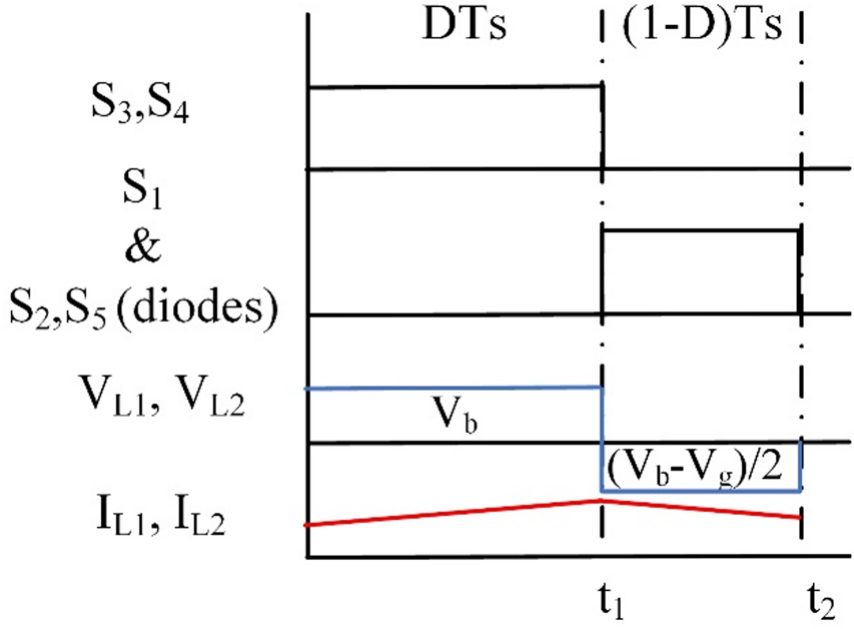
Wave forms of BBB operation in mode 3.

The steady-state waveforms are presented in Fig. 4.

**Fig. 5:**
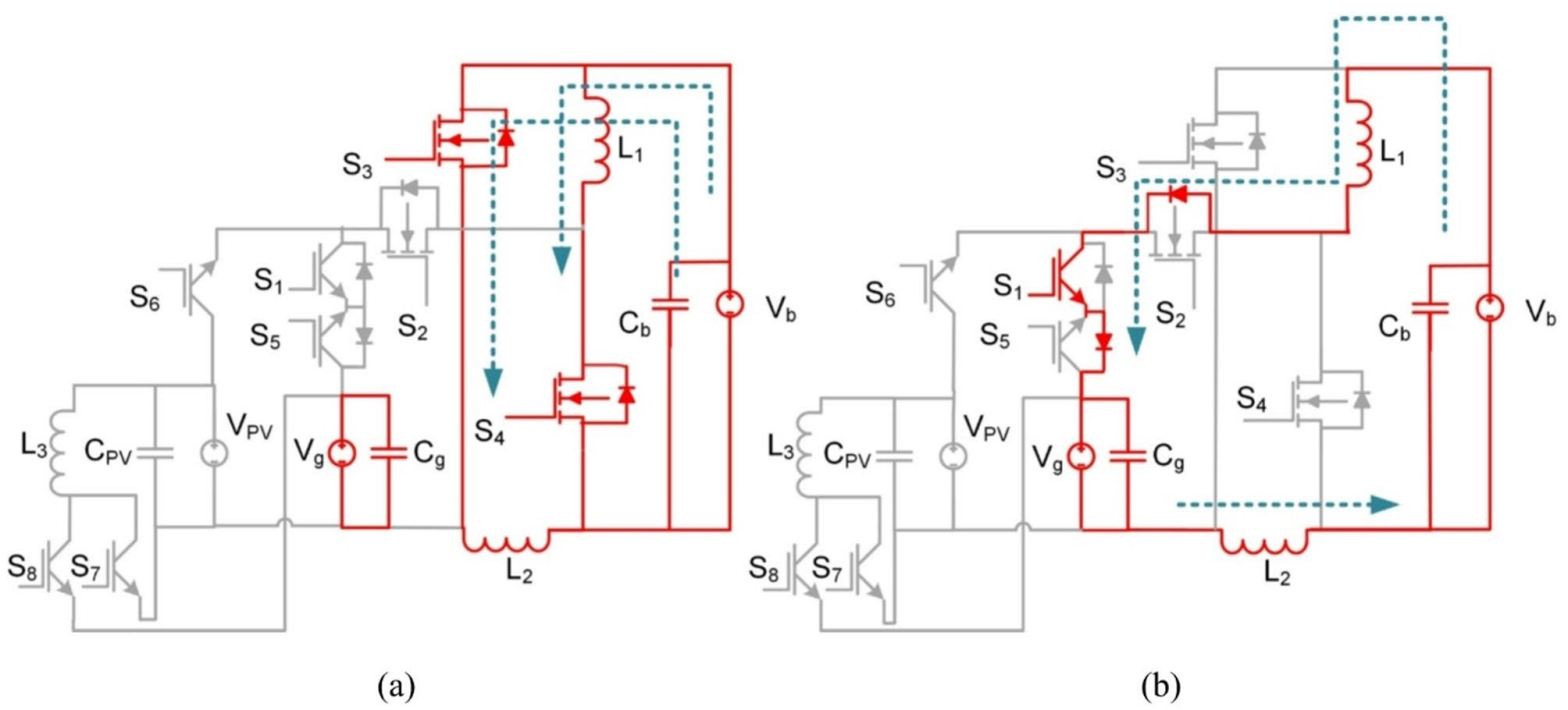
BBB converter operation in mode 3 (**a**) Interval T_i1_, (**b**) Interval T_i2_.

Similarly as modes 1 and 2, at T_i2_: S_3_ and S_4_ are on, and the rest of the switches are off. The battery (V_b_) charges L_1_ and L_2_. The circuit and its operation are shown in Fig. 5(a). Equation (4) is the mathematical representation of the voltages across inductors L_1_ and L_2_.

4 $$\:{V}_{L1}={V}_{L2}={V}_{b}$$

Subsequently at T_i2_: S_1_ and the diodes of S_2_ and S_5_ are on and the rest of the switches are off. A discharge takes place for L_1_ to the grid as shown in Fig. 5(b). Voltages of inductors L_1_ and L_2_ are expressed as follows:

5 $$\:{V}_{L1}={V}_{L2}=\frac{{V}_{b}-{V}_{g}}{2}$$

The gain equation in mode 3 (step-up) is obtained as:

6 $$\:\frac{{V}_{g}}{{V}_{b}}=\frac{1+D}{1-D}$$

#### Fourth mode (step-up) of operation

**Fig. 6:**
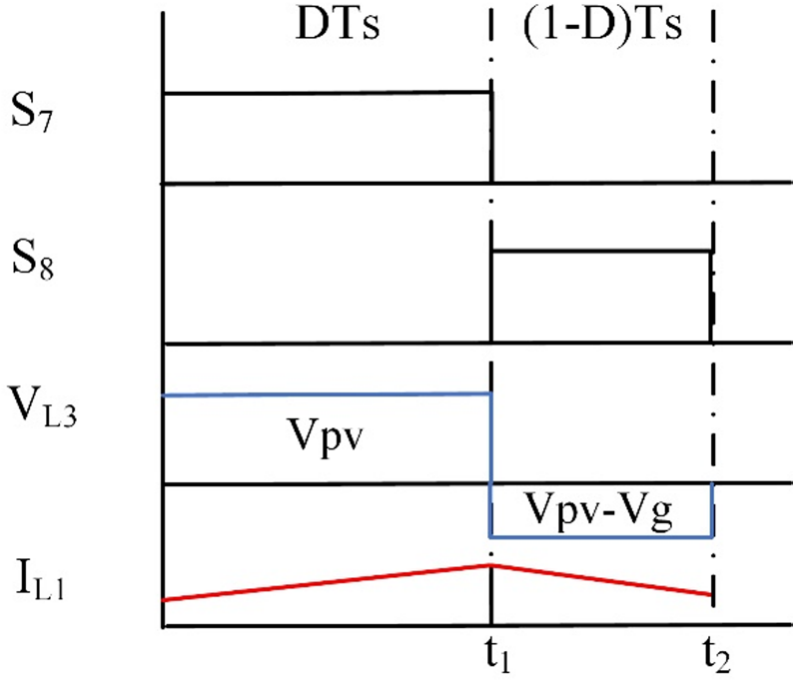
Wave forms of BBB operation in mode 4.

Similarly for the three previous modes. The working intervals waveform are shown in Fig. 6.

**Fig. 7:**
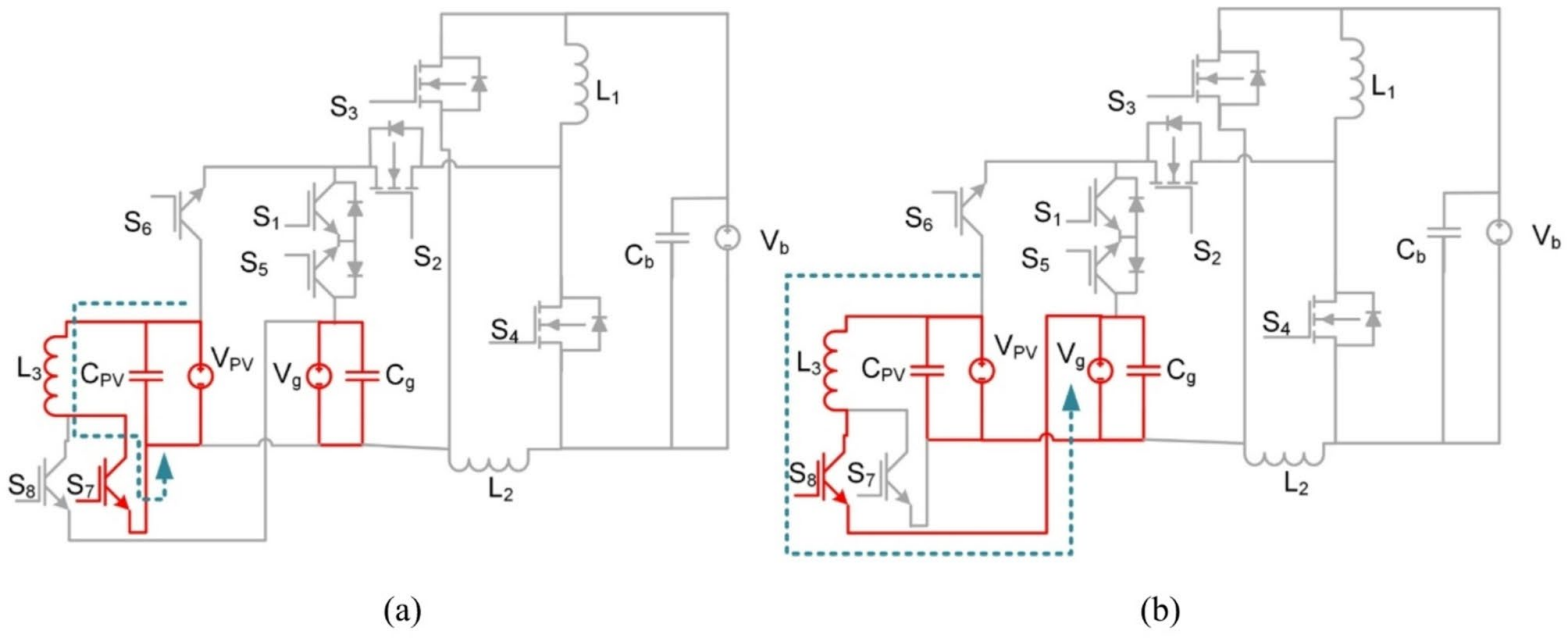
BBB converter operation in mode 4 (**a**) Interval T_i1_, (**b**) Interval T_i2_.

S_7_ is on and the rest of the switches are off, at T_i1_. The energy is stored in inductor L_3_ from the first voltage source (V_pv_) as shown in Fig. 7(a). The voltages across L_3_ inductor is obtained as:

7 $$\:{V}_{L3}={V}_{pv}$$

S_8_ is on, and the rest of the switches are off. In the subsequent interval T_i2_. The energy is delivered to the grid by a series connection of the inductor L_3_ and the PV source as shown in Fig. 7(b). The voltages across L_3_ inductor is obtained as:

8 $$\:{V}_{g}={V}_{pv}+{V}_{L3}$$

The gain of BBB converter in mode 4 (step-up) is obtained using the volt-second balance equations of L_1_ and L_2_ inductors using the following equations:

9 $$\:\frac{{V}_{g}}{{V}_{pv}}=\frac{1}{1-D}$$

### Boost-boost-SEPIC

**Fig. 8:**
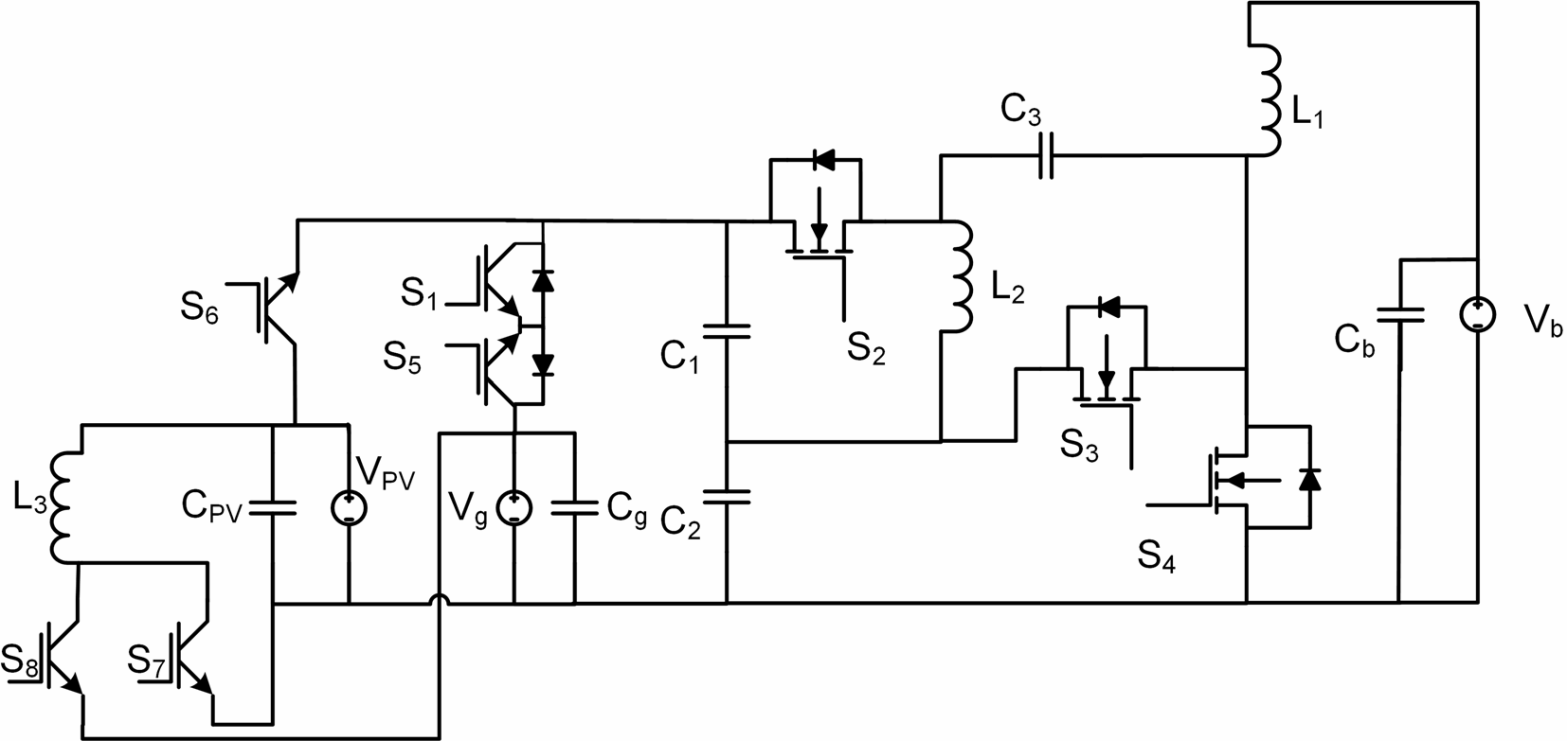
Boost-boost-SEPIC converter circuit.

Based on a combination between boost and single-ended primary-inductor converter (SEPIC), the proposed Boost-Boost-SEPIC (BBS) MPB is configured. The proposed converter uses three MOSFETs (S_2_, S_3_ and S_4_), five IGBTs (S_1,_ S_5,_ S_6,_ S_7_ and S_8_), three inductors (L_1_, L_2_, L_3_), three capacitors (C_1_, C_2_, C_3_), and also as BBB three capacitors are used as filter (C_PV_, C_g_, C_b_), as shown in Fig. 8. The same time intervals as BBB converter are used.

#### First and second modes (step-down) of operation

**Fig. 9:**
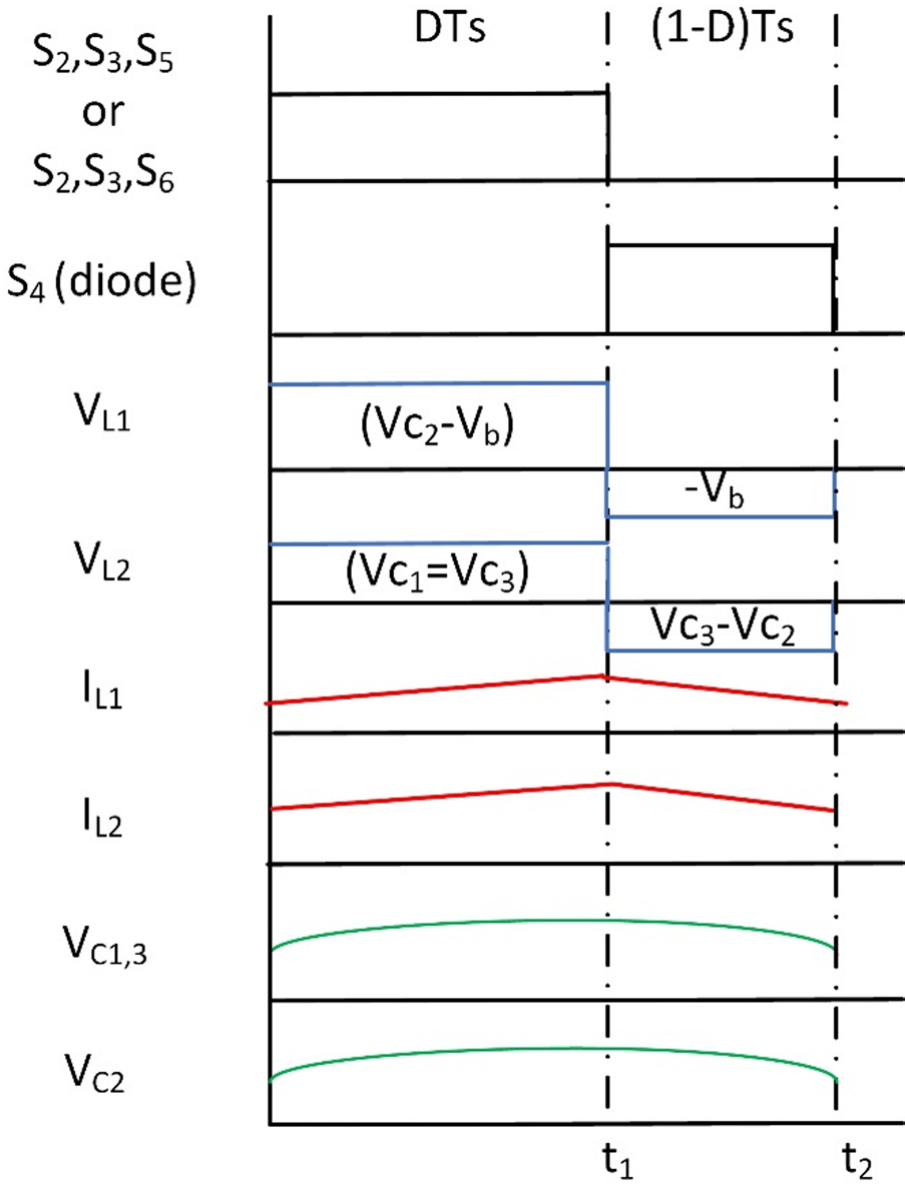
Wave forms of BBS operation in modes 1 and 2.

Similar to BBB-MPB the waveforms of BBS are presented in Fig. 9.

**Fig. 10:**
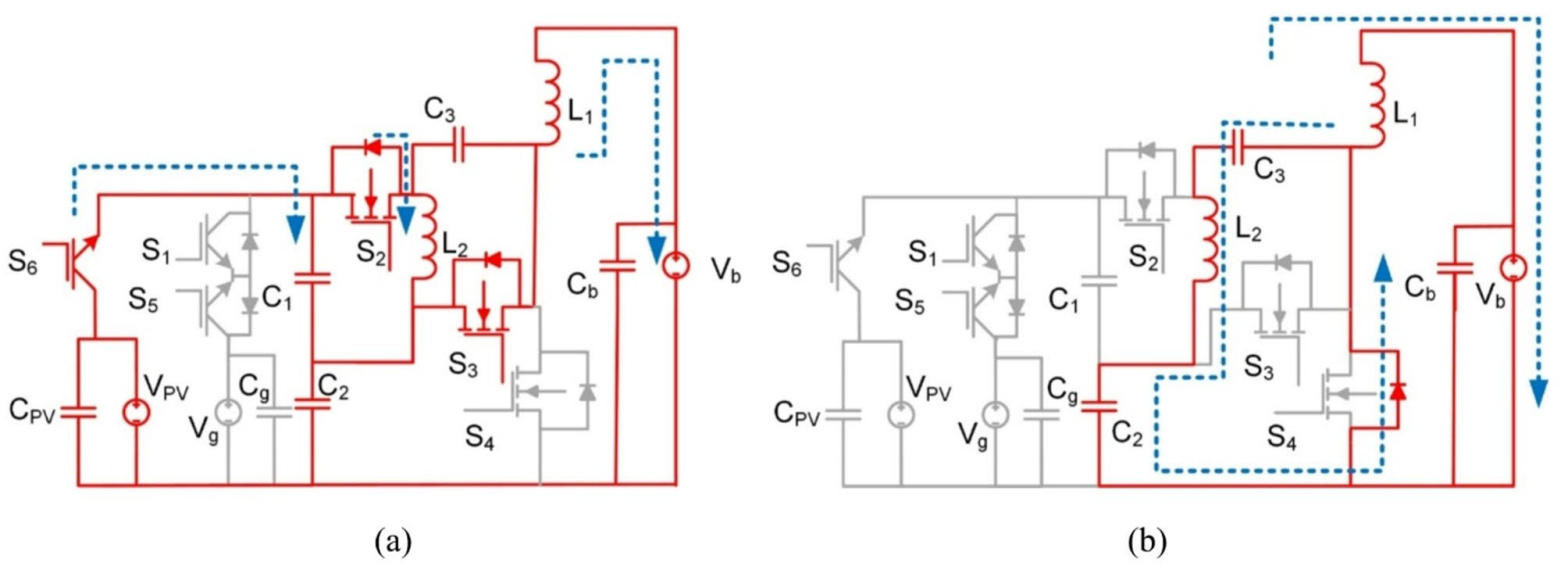
BBS converter operation in modes 1 and 2 (**a**) Interval T_i1_, (**b**) Interval T_i2_.

L_3_, S_7_, and S_8_ are removed from Fig. 10 for illustration purposes to make the figure clearer. As L_3_, S_7_, and S_8_ are only used in mode 4.

At T_i1_: S_2_, S_3_, S_6_ for PV2V mode (S_2_, S_3_, S_5_ for G2V mode) are on, and the rest of the switches are off. The First source (V_pv_ or V_g_) charges L_1_ and L_2_ and capacitors C_1_, C_2_ and C_3_. The circuit and its operation are shown in Fig. 10(a). The following equation is the expression for the voltages of the inductors L_1_ and L_2_.

10 $$\:{V}_{L1}={V}_{C2}-{V}_{b},\:{V}_{C1}={V}_{C3}={V}_{L2}\:$$

At T_i2_: S_4_ diode is on, and the rest of the switches and diodes are off L_1_, L_2_, C_2_ and C_3_ discharges to the load as shown in Fig. 10(b). Voltages of inductors L_1_ and L_2_ are expressed as follows:

11 $$\:{V}_{L1}={V}_{L2}=-{V}_{b}$$

The gain of BBS in both modes 1 and 2 (step-down) are obtained as:

12 $$\:\frac{{V}_{b}}{{V}_{pv}}=\frac{D}{2-D}$$

#### Third mode (step-up) of operation

**Fig. 11:**
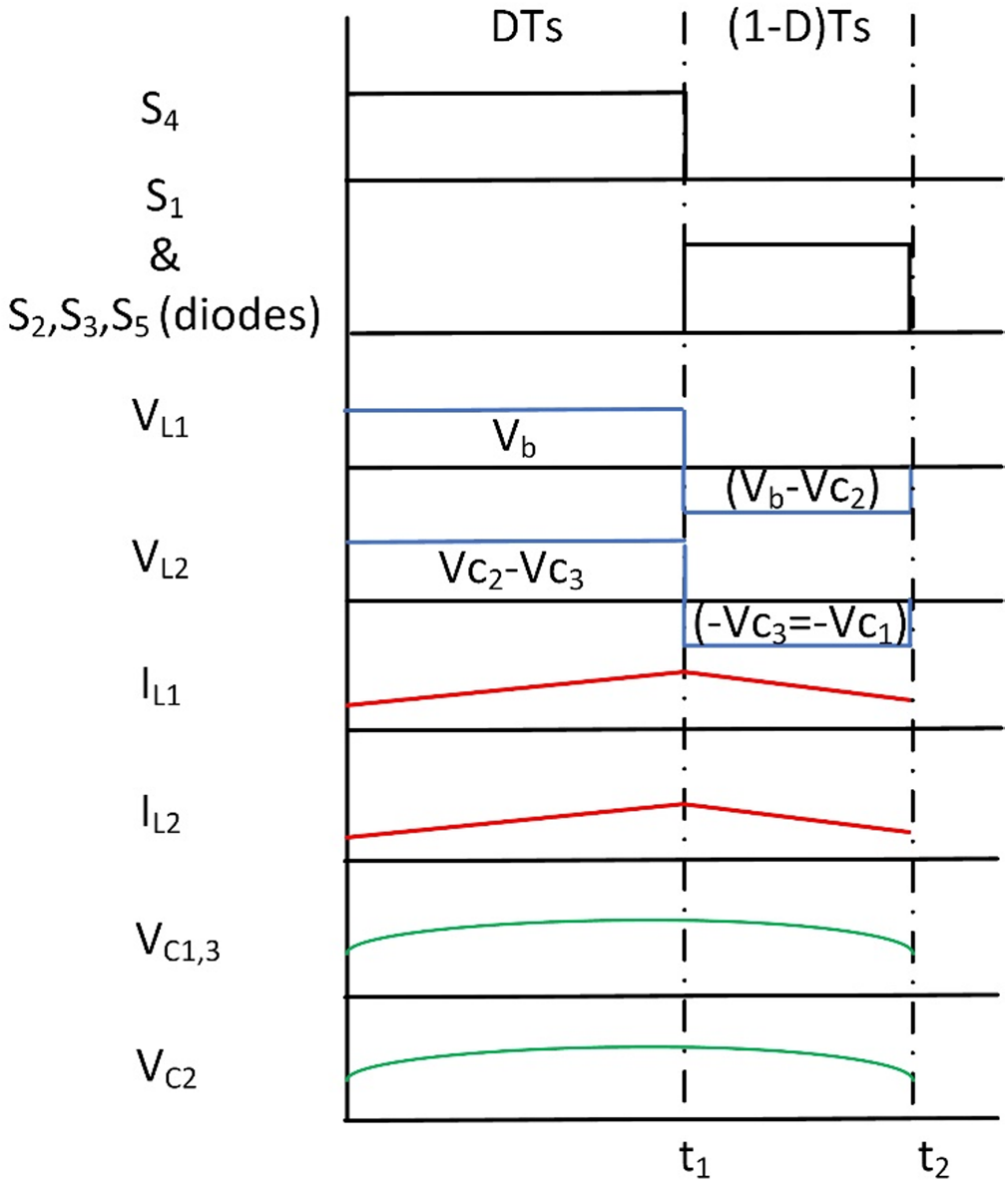
Wave forms of BBS operation in mode 3.

The steady-state analytical waveforms in mode 3 for the BBS converter are presented in Fig. 11.

**Fig. 12:**
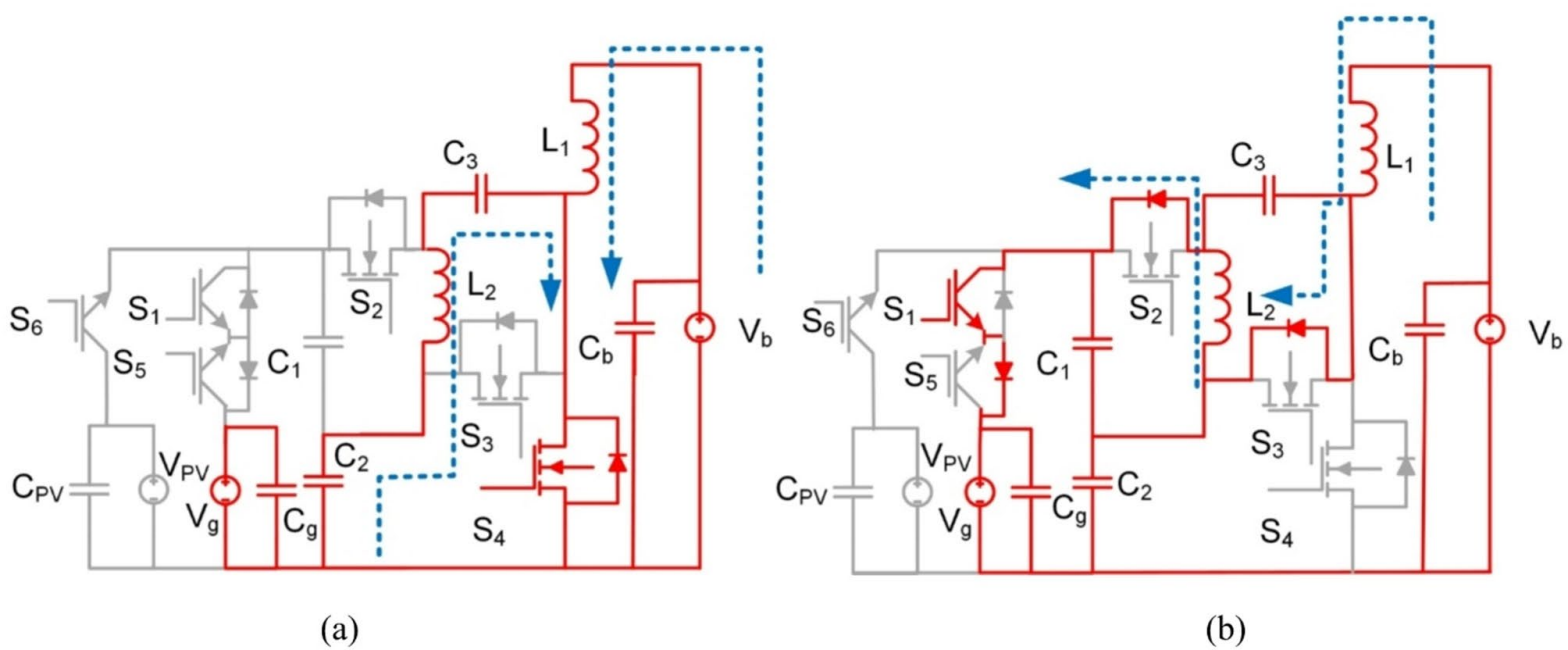
BBS converter operation in mode 3 (**a**) Interval T_i1_, (**b**) Interval T_i2_.

L_3_, S_7_, and S_8_ are removed from Fig. 12 for illustration purposes to make the figure clearer. As L_3_, S_7_, and S_8_ are only used in mode 4.

Similar to mode 3 in BBB, at T_i1_: S_4_ is on, and the rest of the switches and diodes are off. The battery (V_b_) charges L_1_ and L_2_. The equivalent circuit and its operation are shown in Fig. 12(a) the voltages of the inductors L_1_, L_2_ and the grid voltage are expressed as follows:

13 $$\:{V}_{L1}={V}_{b}$$

14 $$\:{V}_{L2}={V}_{C2}-{V}_{C3}$$

15 $$\:{V}_{g}={V}_{C1}+{V}_{C2}$$

In the subsequent time interval T_i2_: S_1_ along with diodes of S_2_, S_3_, and S_5_ are turned on and the rest of the switches are off. A discharge takes place for L_1_ to the grid as shown in Fig. 12(b). Voltages of inductors L_1_, L_2_ and grid are expressed as follows:

16 $$\:{V}_{L1}={V}_{b}-{V}_{C2}$$

17 $$\:{V}_{L2}=-{V}_{C3}=-{V}_{C1}$$

18 $$\:{V}_{g}={V}_{C1}+{V}_{C2}$$

The gain of BBS in mode 3 (step-up) is obtained as:

19 $$\:\frac{{V}_{g}}{{V}_{b}}=\frac{1+D}{1-D}$$

For both converters BBB and BBS, the fourth mode of operation is the same. So the same theoretical formulas are applied.

### DCM and BCM for BBB-MPB

#### BCM for BBB-MPB

**Fig. 13:**
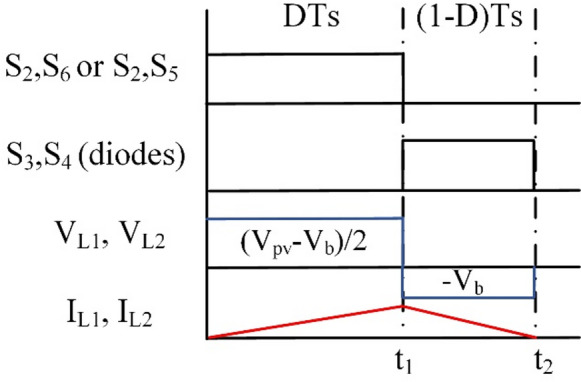
Wave forms of BBB operation in mode 1 under BCM.

The BBB converter operates at the BCM between CCM and DCM, where the inductor current reaches zero exactly at the end of the switching cycle as illustrated in Fig. 13.

For modes 1 and 2 the key equations are (1) and (2). The inductor peak current can be calculated using the following equations.

At T_i1_ the peak current can be obtained as:

20 $$\:{I}_{Peak}=\frac{{(V}_{pv}-{V}_{b})D{T}_{s}}{2L}$$

At T_i2_ current falls to zero and is calculated as:

21 $$\:{I}_{o}=\frac{{V}_{b}(1-D){T}_{s}}{L}$$

By simplifying the equations, an expression of the gain is obtained.

22 $$\:{I}_{Peak}=\frac{{(V}_{pv}-{V}_{b})D{T}_{s}}{2L}=\frac{{V}_{b}(1-D){T}_{s}}{L}$$

23 $$\:\frac{{V}_{b}}{{V}_{pv}}=\frac{D}{2-D}$$

The same gain as CCM is obtained. To calculate the critical inductance of modes 1, and 2 the following equations can be used.

24 $$\:{I}_{b}=\frac{{V}_{b}}{R}=\frac{{I}_{Peak}(1-D){T}_{s}}{{T}_{s}}$$

By substituting I_Peak_ an expression of the critical inductance can be obtained.

25 $$\:{L}_{Crit\:1,\:\:2}=\frac{{V}_{b}{(1-D)}^{2}{T}_{s}}{{I}_{b}}$$

Same analysis is done to obtain the critical inductance for modes 3 and 4.

26 $$\:{L}_{Crit\:3}=\frac{{V}_{g}{(1-D)}^{2}{T}_{s}}{{2I}_{g}}$$

27 $$\:{L}_{Crit\:4}=\frac{{V}_{g}{(1-D)}^{2}{T}_{s}}{{I}_{g}}$$

#### DCM for BBB-MPB

**Fig. 14:**
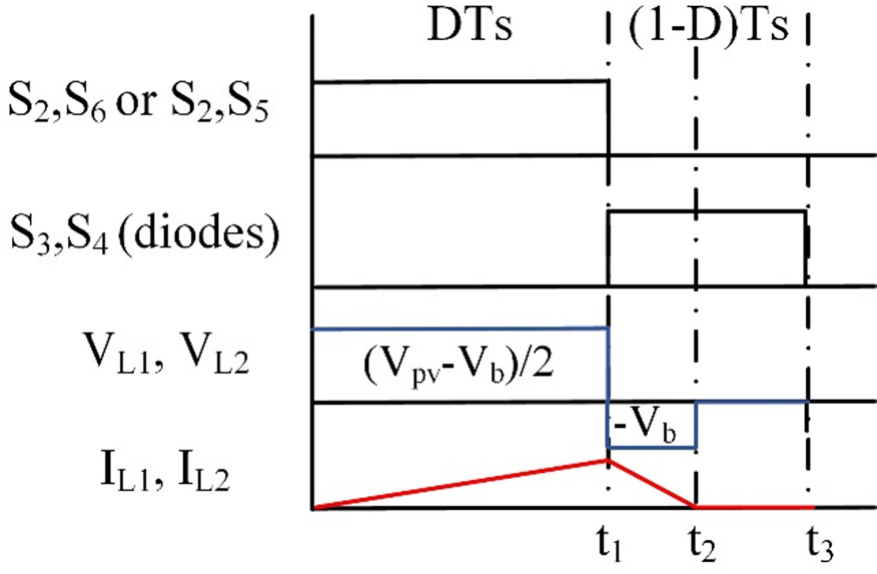
Wave forms of BBB operation in mode 1 under DCM.

The BBB converter operates at the DCM, where the inductor current reaches zero before the end of the switching cycle and stays zero till the end of the cycle as illustrated in Fig. 14. In DCM there are 3 intervals T_i1_ (t_0_ – t_1_), T_i2_ (t_1_ – t_2_), and T_i3_ (t_2_ – t_3_). T_i1_ is the interval where the inductor is charging, T_i2_ is the discharging interval, T_i3_ is the zero current interval.

At T_i1_ the inductor voltage is the same as Eq. (1), while in interval T_i2_ the inductor voltage can be calculated as Eq. (2). However at T_i3_ the inductor voltage is zero. The peak current at the end of the first interval is obtained as mentioned in Eq. (20). The current decreases over the second interval until it is zero as follows:

28 $$\:{I}_{L}={I}_{Peak}-\frac{{V}_{b}\:{T}_{i2}}{L}$$

At the third time interval I_L_ is zero. Using volt-second balance equation an expression of the output voltage is obtained as:

29 $$\:{V}_{b}={V}_{pv}\frac{D}{D+2k}$$

Where k can be obtained from the output charge balance Eq. 

30 $$\:\frac{{V}_{b}}{R}=\frac{{I}_{Peak}k\:{T}_{s}}{2{T}_{s}}$$

31 $$\:k=\frac{{2V}_{b}}{{I}_{PeakDisplay\_Equation\_num}R}$$

Finally the voltage gain and critical inductance can be obtained as:

32 $$\:{M}_{\text{1,2}}=\frac{{D}^{2}}{{D}^{2}+\frac{4L}{R{T}_{s}}}$$

33 $$\:{L}_{Crit\:1,\:\:2}=\frac{R{(1-D)}^{2}{T}_{s}}{4}$$

Similarly mode 3 can be calculated as mode 1. Hence the voltage gain and critical inductance of mode 3 are obtained.

34 $$\:{M}_{3}=\frac{1}{2}(1+\sqrt{1+\frac{{8D}^{2}R{T}_{s}}{L}})$$

35 $$\:{L}_{Crit\:3}=\frac{R{(1-D)}^{2}{T}_{s}}{2}$$

And finally for mode 4 the DCM gain and critical inductance are like a normal boost converter.

36 $$\:{M}_{4}=\frac{1}{2}(1+\sqrt{1+\frac{{2D}^{2}R{T}_{s}}{L}})$$

37 $$\:{L}_{Crit\:4}=\frac{R{(1-D)}^{2}{T}_{s}}{2}$$

## Non-Ideal analysis, voltage stress and current stress of BBB-MPB converter

### Voltage and current ripples

As stated in^22^ the voltage and current ripple of BBB-MPB is same as boost converter as it is the same topology. However, for the BBS-MPB as the capacitors are connected in stack the ripples are half that of the boost converter.

The voltage ripple can be calculated as:

38 $$\:\varDelta\:V=\frac{{V}_{pv,g}\:D}{R\:Fs\:C}$$

Where R is the output resistance, Fs is the switching frequency, and C is the output capacitance.

Furthermore, the current ripple is calculated by the subsequent equation. And it is also the same as boost converter as the connection of the inductors are the same as boost converter.

39 $$\:\varDelta\:I=\frac{{V}_{b}\:D}{Fs\:L}$$

In mode 4 (step-up), the converter is working as a normal boost converter so the above equations can be applied for this mode.

### Gain analysis for a non-ideal BBB-MPB converter

In this section only BBB-MPB converter with parasitic elements is studied as it has higher efficiency and lower number of components which in fact produce lower losses. To obtain the output voltages in each mode parasitic elements where added to the BBB-MPB model, where r_S1_, r_S2_, r_S3_, r_S4_, r_S5_, r_S6_ are the on-state resistance of S_1_, S_2_, S_3_, S_4_, S_5_, S_6_. r_L1_, r_L2_ and r_L3_ are the resistances of L_1_, L_2_ and L_3_ respectively, r_d_ is the diode resistance and R is the output resistance^22,23^.

When attempting to determine the output voltage of modes 1 and 2, analysis is carried as previous sections and the output is observed in Eq. (40):

40 $$\:{V}_{b}=\frac{{V}_{pv,g}D}{\frac{2\left({r}_{S3}+{r}_{S6+}{r}_{d3}+{r}_{d4}+{r}_{L1}+{r}_{L2}\right)\left[1+D\right]}{DR}+(2-D)}$$

For mode 3 the output voltage is obtained as:

41 $$\:{V}_{g}=\frac{{V}_{b}(1+D)}{{({r}_{s1}+{r}_{d5}+{r}_{d2}+r}_{s3}+{r}_{s4}+{r}_{L1}+{r}_{L2})\left[\frac{2+D}{R}\right]+(1-D)}$$

Finally, for mode 4 the output voltage is obtained as:

42 $$\:{V}_{g}=\frac{{\text{V}}_{\text{p}\text{v}\:}\left(1-\text{D}\right)\text{R}}{{(1-\text{D})}^{2}\text{R}+{\text{r}}_{\text{L}3}+\text{D}{r}_{on,S7}+{r}_{on,S8}(1-\text{D})}$$

### Voltage stress analysis

The voltage stress across each switching device is obtained for each mode of the four modes of the BBB-MPB and BBS-MPB using the circuit analysis in the previous section. The maximum voltage across MOSFETs and IGBTs is illustrated in Table 1^22,23^.

**Table 1:** MOSFETs and IGBTs voltage stresses for BBB and BBS MPB Converters.

BBB-MPB	S_1_, S_5_,S_6_	S_2_	S_3_, S_4_	S_7_	S_8_
	$\:{V}_{PV}+{V}_{g}$	$\:{V}_{g}+{V}_{b}$	$\:{V}_{PV,g}+{V}_{b}/2$	$\:{{V}_{CE}+V}_{g}$	$\:{V}_{CE}-{V}_{g}$

BBS-MPB	S_1_, S_5_, S_6_	S_2_, S_3_	S_4_	S_7_	S_8_
	$\:{V}_{PV}+{V}_{g}$	${V}_{b}/1-D$	$\:{V}_{b}/1-D$	$\:{{V}_{CE}+V}_{g}$	$\:{V}_{CE}-{V}_{g}$

### Current stress analysis

The current stress on MOSFETs and IGBTs in each mode of the BBB and BBS MPB converters can be determined by using the equivalent circuits for each mode. The maximum current across MOSFETs and IGBTs is illustrated in Table 2^22,23^.

**Table 2:** MOSFETs and IGBTs current stresses for BBB and BBS Converters.

BBB-MPB Mode	S_1_,S_5_, S_2_	S_3_, S_4_	S_7_	S_8_
1	$\:{I}_{S}={-I}_{PV}$	$\:{I}_{S}={I}_{PV}/2$	-	-
2	$\:{I}_{S}={-I}_{g}$	$\:{I}_{S}={I}_{g}/2$	-	-
3	$\:{I}_{S}={-I}_{b}$	$\:{I}_{S}={I}_{b}/2$	-	-
4	-	-	$\:{I}_{T1}=(1-D){I}_{g}$	$\:{I}_{T2}={I}_{g}$

BBS-MPB Mode	S_1_,S_5_, S_2_	S_3_, S_4_	S_7_	S_8_
1	$\:{I}_{S}={(-2/2-D)I}_{b}$	$\:{I}_{S}={(1/2-D)I}_{b}$	-	-
2	$\:{I}_{S}={(-2/2-D)I}_{b}$	$\:{I}_{S}={(1/2-D)I}_{b}$	-	-
3	$\:{I}_{S}=2\:{I}_{g}/(1-D)$	$\:{I}_{S}=-\:{I}_{g}/(1-D)$	-	-
4	-	-	$\:{I}_{T1}=(1-D){I}_{g}$	$\:{I}_{T2}={I}_{g}$

## Results and validation

### Theoretical and simulation results

**Fig. 15:**
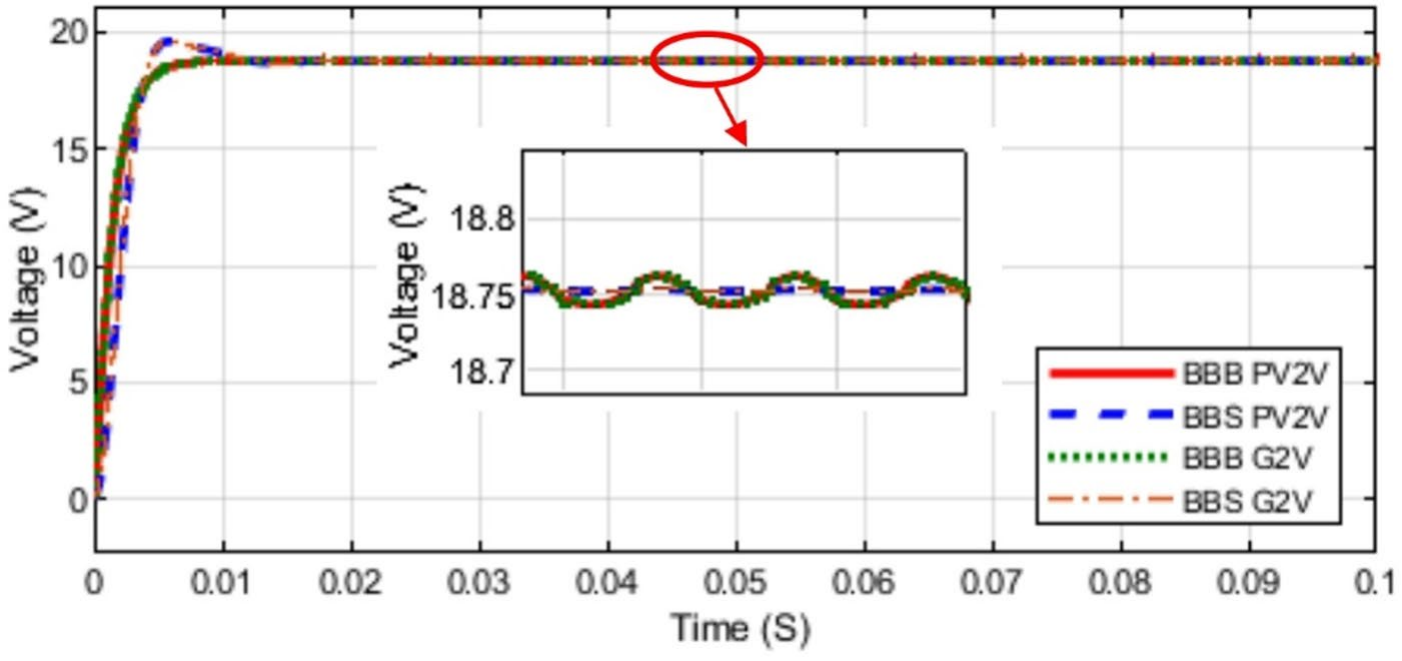
Modes 1 and 2 (PV2V and G2V Voltages) Simulation for BBB and BBS Converters.

**Fig. 16:**
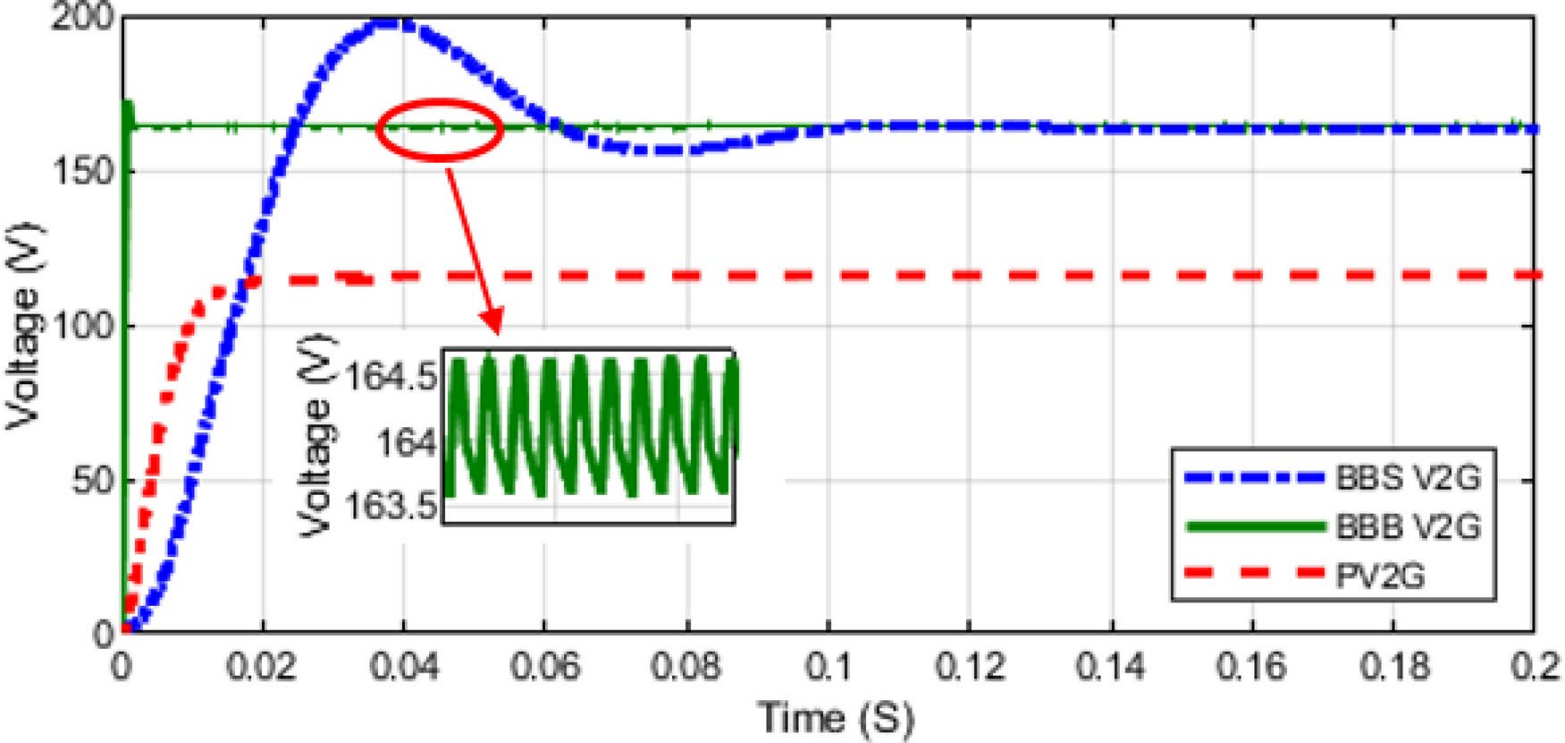
Modes 3 and 4 (V2G and PV2G Voltages) Simulation for BBB and BBS Converters.

This section presents a comparison of theoretical and simulation results for BBB and BBS MPBs. Simulation tests were conducted on the two converters for modes 1, 2, and 3. The input voltage for each mode is 56.5 V. The output voltage for modes 1, 2, and 3 is observed in Figs. 15 and 16. The MPBs undergo testing with duty cycles ranging from 10% to 90% to quantify the error between theoretical and simulated results. In addition, if the error increases, the losses increases so the efficiency decreases.

In order to verify the theoretical analysis of the suggested converters, a simulation is conducted using MATLAB/SIMULINK. Table 3 shows the components and their corresponding specs.

**Fig. 17:**
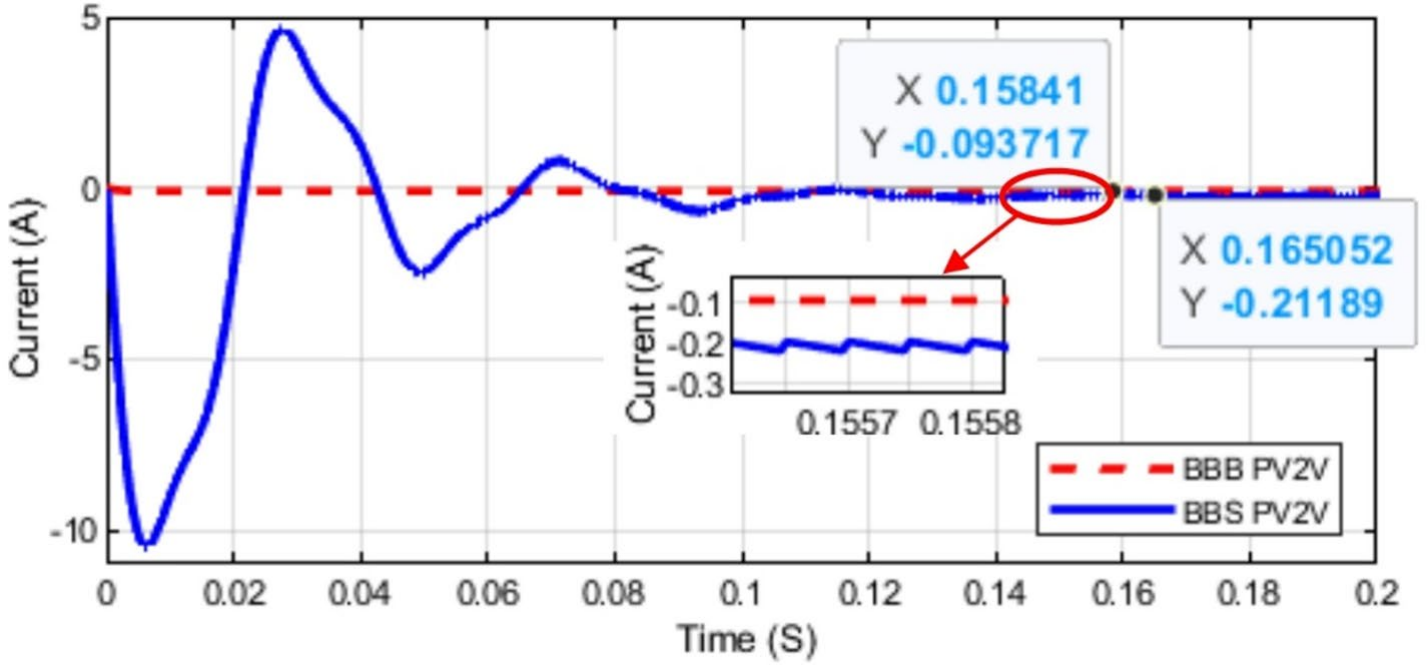
Mode 1 (PV2V Current) Simulation for BBB and BBS Converters.

The simulation results for the BBB and BBS MPBs in all four modes are presented in Figs. 15, 16 and 17. A duty ratio of 50% is used at the gate input of all MOSFETs and IGBTs. The selection of MOSFETs and IGBTs was mostly determined by their availability and cost-effectiveness throughout the prototype development phase. It provides sufficient performance to substantiate the proposed design concept. Nonetheless, this selection is hardly limiting.

The selection of power devices can be customized based on the necessary power ratings, efficiency, and performance criteria in future research or industrial applications.

**Table 3:** Components and their specifications for BBB and BBS converters.

Parameter	Specification
Switching frequency	20 KHz
Switches (MOSFETs and IGBTs) IRFP250 (MOSFETs)	Data sheet: r_on_=0.085 Ω, I_Dmax_.=33 A, V_GS_=12 V. Operating point: r_on_=0.085 Ω, I_D_=18 A, V_GS_=12 V, V_DS_=150 V.
GT35J321 (IGBTs)	Data sheet: V_Cmax_.=600 V, I_Cmax_.=18 A, I_Fmax_.=20 A, V_CE_=2.3 V, V_F_=2 V. Operating point: V_C_.=400 V, I_C_=12 A, I_F_=12 A, V_CE_=2.3 V, V_F_=2 V.
IRGB15B60KD (IGBTs)	Data sheet: V_Cmax_.=600 V, I_Cmax_.=30 A, V_CE_=2 V. Operating point: V_C_.=150 V, I_C_=18 A, V_CE_=2 V.
Capacitors	220 uF
Inductors	200 uH, 0.12 Ω
PV, grid and battery voltages	56.5 V
R (load resistance)	20 Ω

As observed from the above figures the simulation results comply with the theoretical analysis. At a duty cycle of 50% the output of BBB and BBS MPBs in modes 1, 2, 3, and 4 are shown in Figs. 15 and 16. Mode 1 current is shown in Fig. 17. The output of mode 4 is the same for both BBB and BBS as it is the same topology. Simulation results indicate that mode 3 of BBB converter exhibits lower losses. In mode 1 and 2, the BBB-MPB exhibits a slightly higher level of efficiency. In addition, the BBB-MPB converter is built with less components compared to the BBS-MPB.

### Practical results for BBB-MPB

**Fig. 18:**
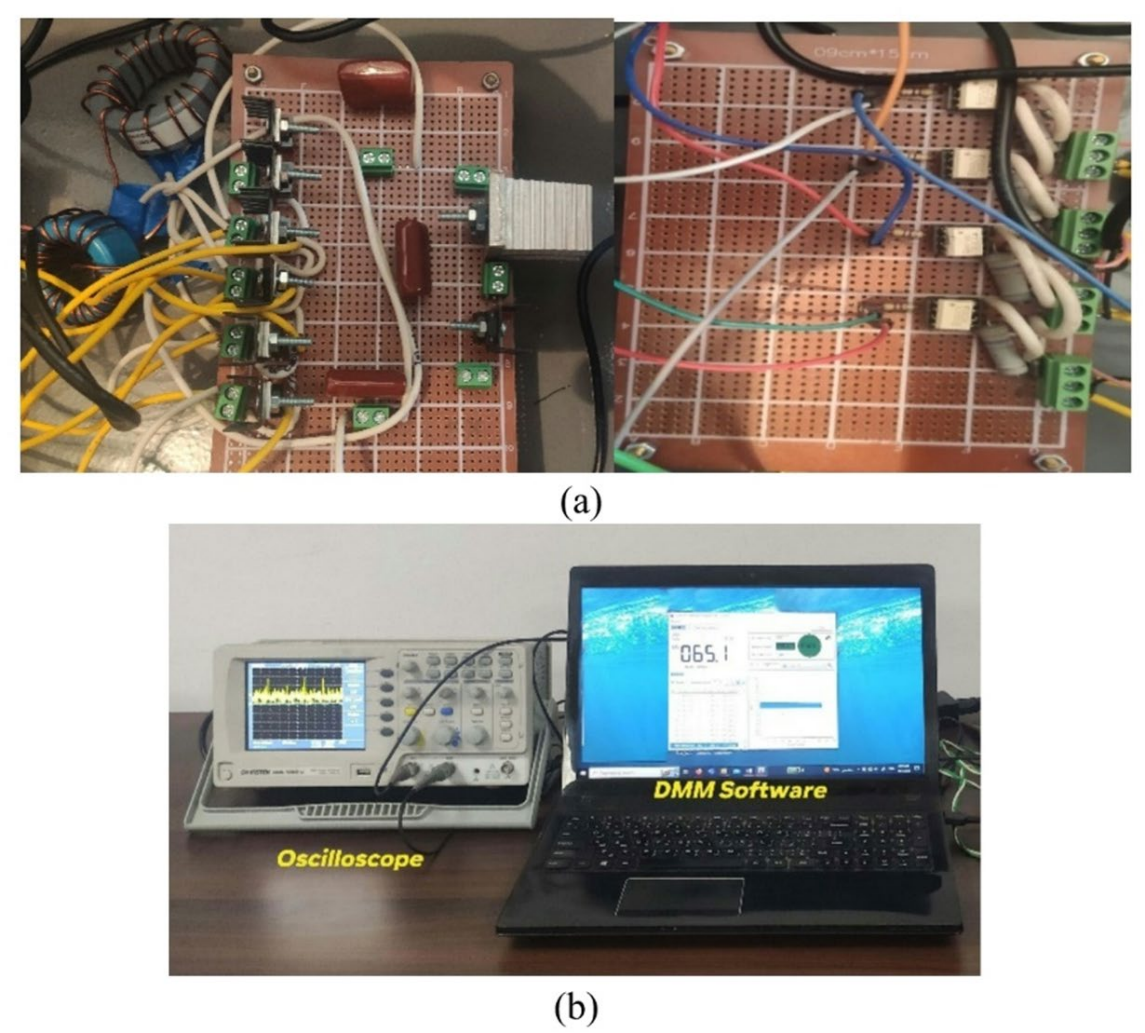
(**a**) BBB-MPB and driver circuit; (**b**) test bench.

The BBB-MPB prototype is tested utilizing non-ideal components as outlined in Table 3. The prototype undergoes testing in all four modes, and the experimental setup is shown in Fig. 18. The test bench consists of the BBB prototype, gate driver circuit to drive the switches, oscilloscope to display the waveforms, DMM with its software to measure the output voltage and currents. The oscilloscope probe was set to x10 to divide the voltage by 10, as the maximum range is 10 V/div.

**Fig. 19:**
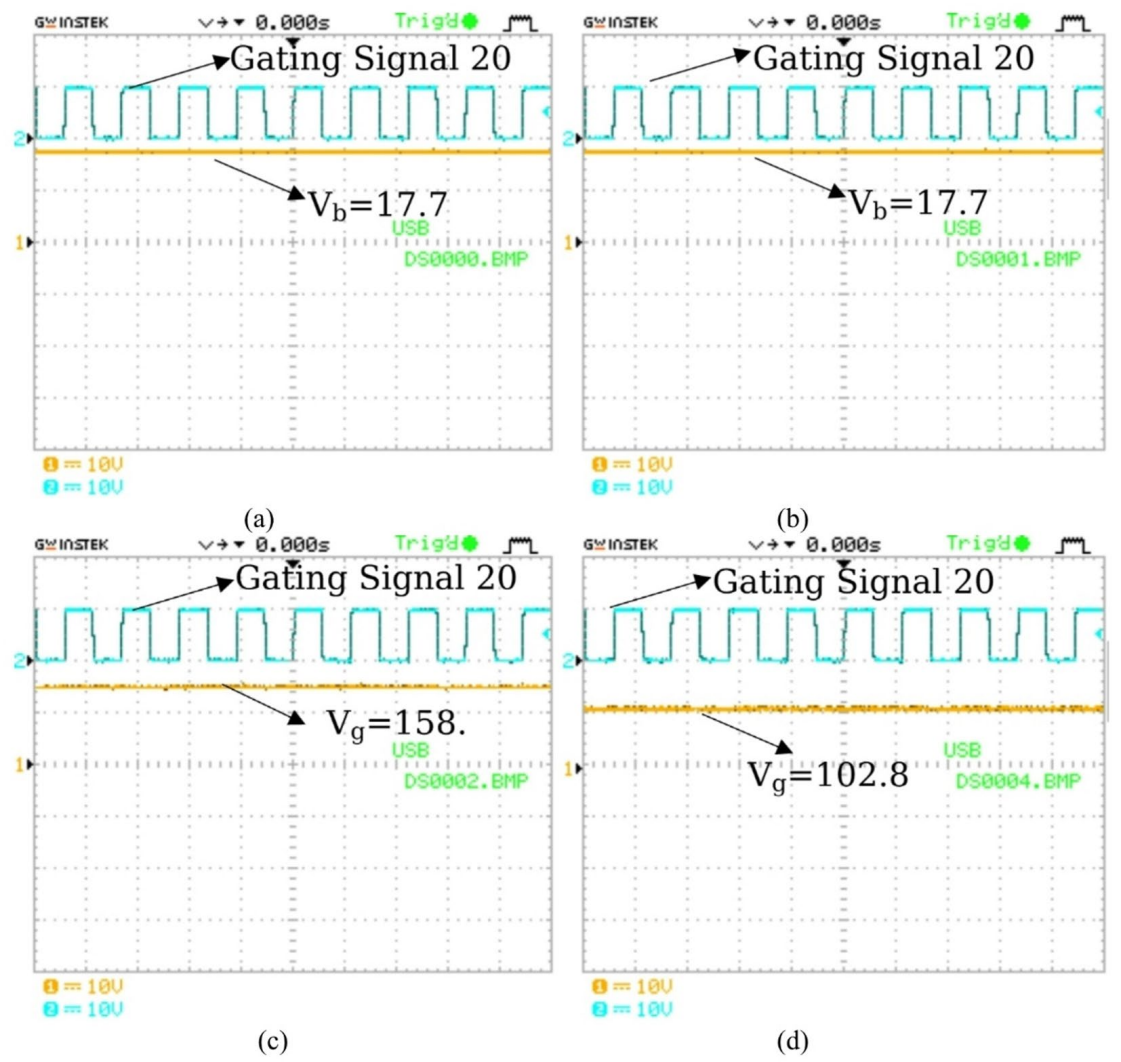
Practical output voltage and gating signal: (**a**) PV2V; (**b**) G2V; (**c**) V2G; (**d**) PV2G.

**Fig. 20:**
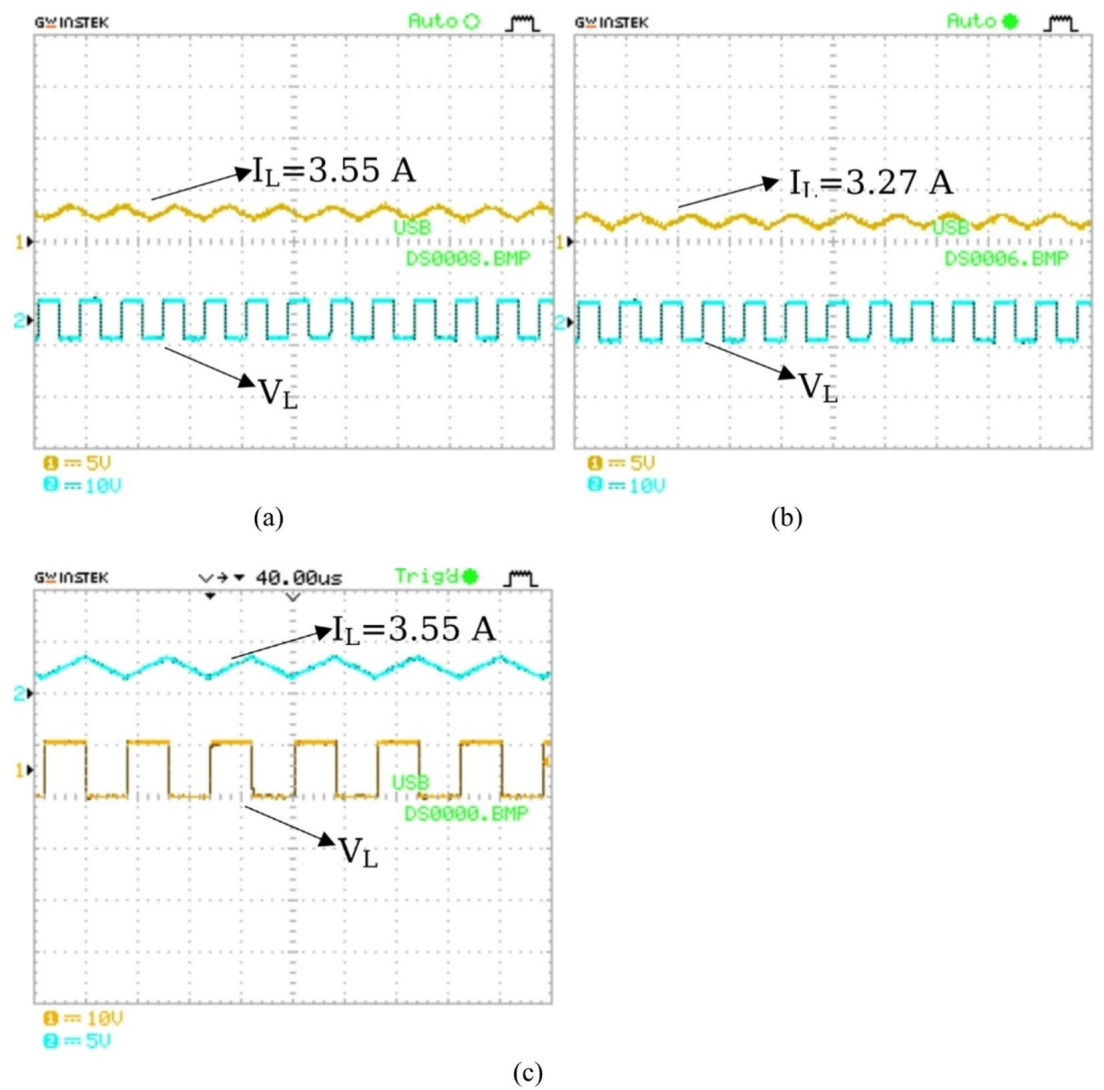
Inductor current and voltage measurements: (**a**) Mode 1 (PV2V); (**b**) Mode 3 (V2G); (c) Mode 4 (PV2G).

The practical output results are presented in Figs. 19 and 20. BBB is tested under a duty ratio of 50%. Output voltages for modes 1 and 2 are represented in Fig. 19(a) and Fig. 19(b). Figure 19(a) and Fig. 19(b)illustrate the power transfer from either the PV system or the grid to the vehicle, where the vehicle voltage is measured as 17.7 V when the PV or grid voltage is 56.5 V. In contrast, Fig. 19(c) highlights the bidirectional capability, with power flowing from the vehicle back to the grid. In this case, the grid voltage is 158.3 V when the vehicle voltage is 56.5 V. Furthermore, Fig. 19(d) presents the PV2G mode, where the grid voltage reaches 102.83 V with a PV input of 56.5 V. The maximum power is measured to be 62.8 W for modes 1 and 2, 517 W for mode 3, and 365 W for mode 4. The results verify the bidirectional nature of the proposed system, confirming that power can reliably flow in both directions depending on the selected operating mode.

The current flowing through the inductor (L_1_) is measured in both mode 1 and mode 3 as seen in Fig. 20(a) and Fig. 20(b). The current is measured to be 3.55 A for mode1 and 3.27 A for mode 3. The current is then displayed on the oscilloscope. In addition, mode 4 current is measured to be 3.55 A and is demonstrated in Fig. 20(c).

**Fig. 21:**
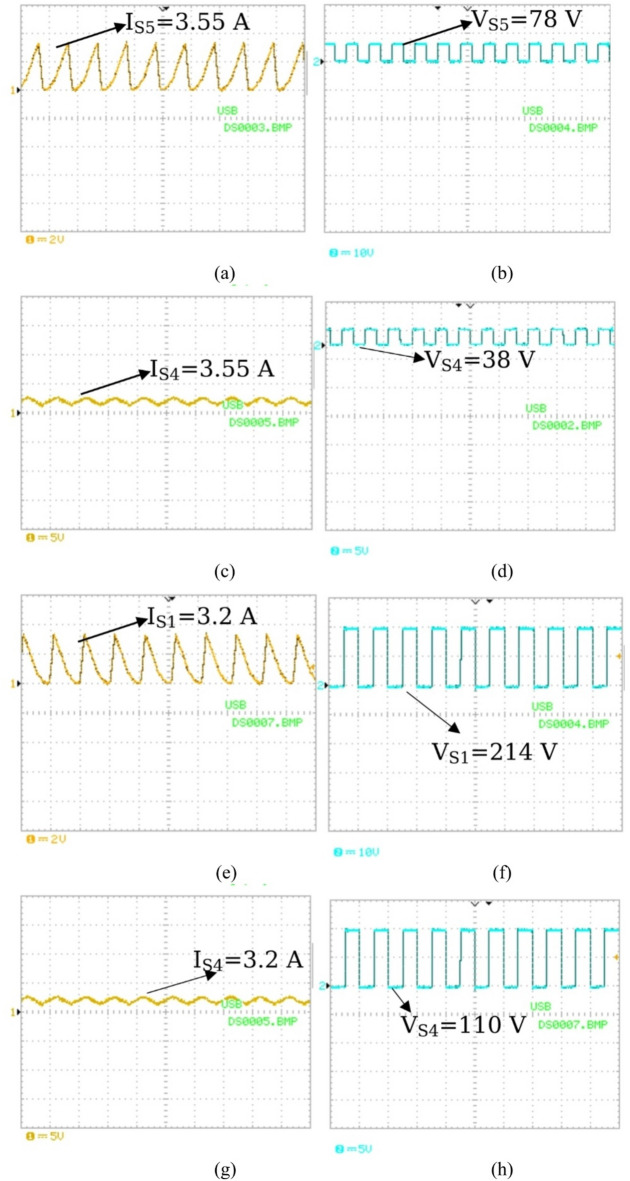
(**a**) Switch S_5_ Current Stress (PV2V); (**b**) Switch S_5_ Voltage Stress (PV2V); (**c**) Switch S_4_ Current Stress (PV2V); (**d**) Switch S_4_ Voltage Stress (V2G); (**e**) Switch S_1_ Current Stress (V2G); (**f**) Switch S_1_ Voltage Stress (V2G); (**g**) Switch S_4_ Current Stress (V2G); (**h**) Switch S_4_ Voltage Stress (V2G).

Figure 21 Illustrates the voltage and current stresses in modes 1 and 3. Figure 21(a) shows the current stress of switch S_5_ in mode 1, Fig. 21(b) shows the voltage stress of switch S_5_ in mode 1, Fig. 21(c) shows the current stress of switch S_4_ in mode 1, Fig. 21(d) shows the voltage stress of switch S_4_ in mode 1.

In addition Fig. 21(e) shows the current stress of switch S_1_ in mode 3, Fig. 21(f) shows the voltage stress of switch S_1_ in mode 3, Fig. 21(g) shows the current stress of switch S_4_ in mode 3, Fig. 21(h) shows the voltage stress of switch S_4_ in mode 3.

**Fig. 22:**
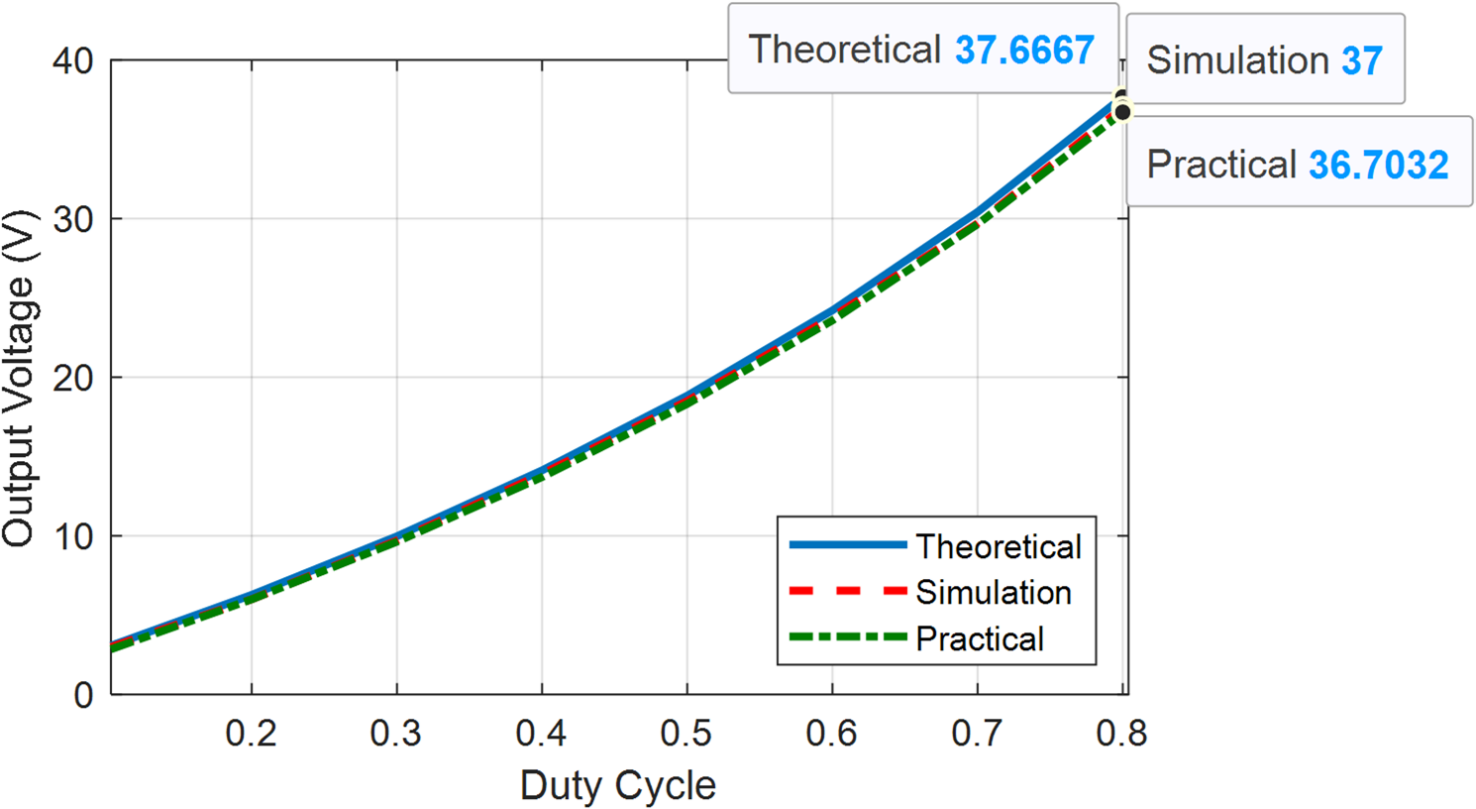
Output voltages vs. duty cycle of BBB-MPB in modes 1 and 2 (theoretical, simulation and practical).

**Fig. 23:**
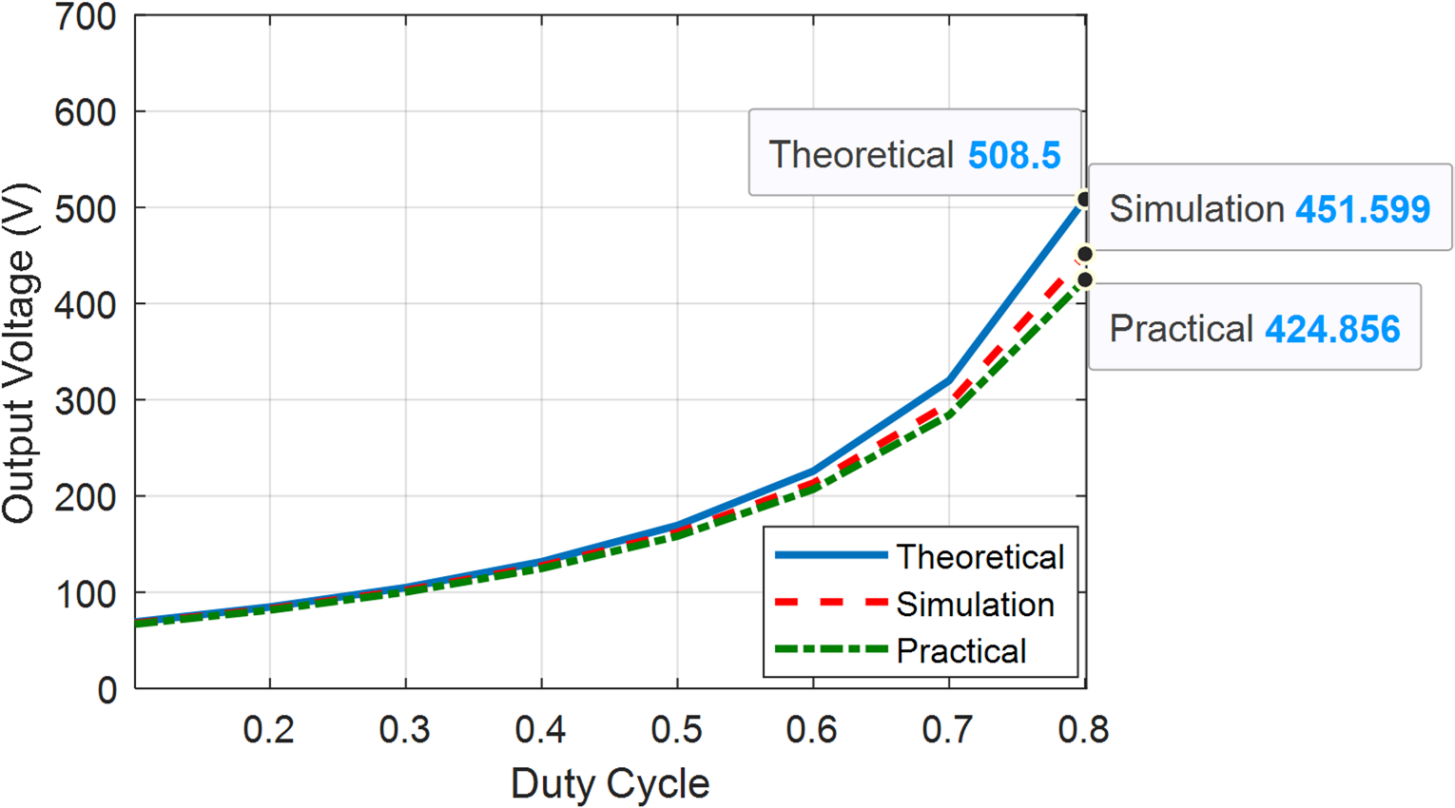
Output voltages vs. duty cycle of BBB-MPB in mode 3 (theoretical, simulation and practical).

**Fig. 24:**
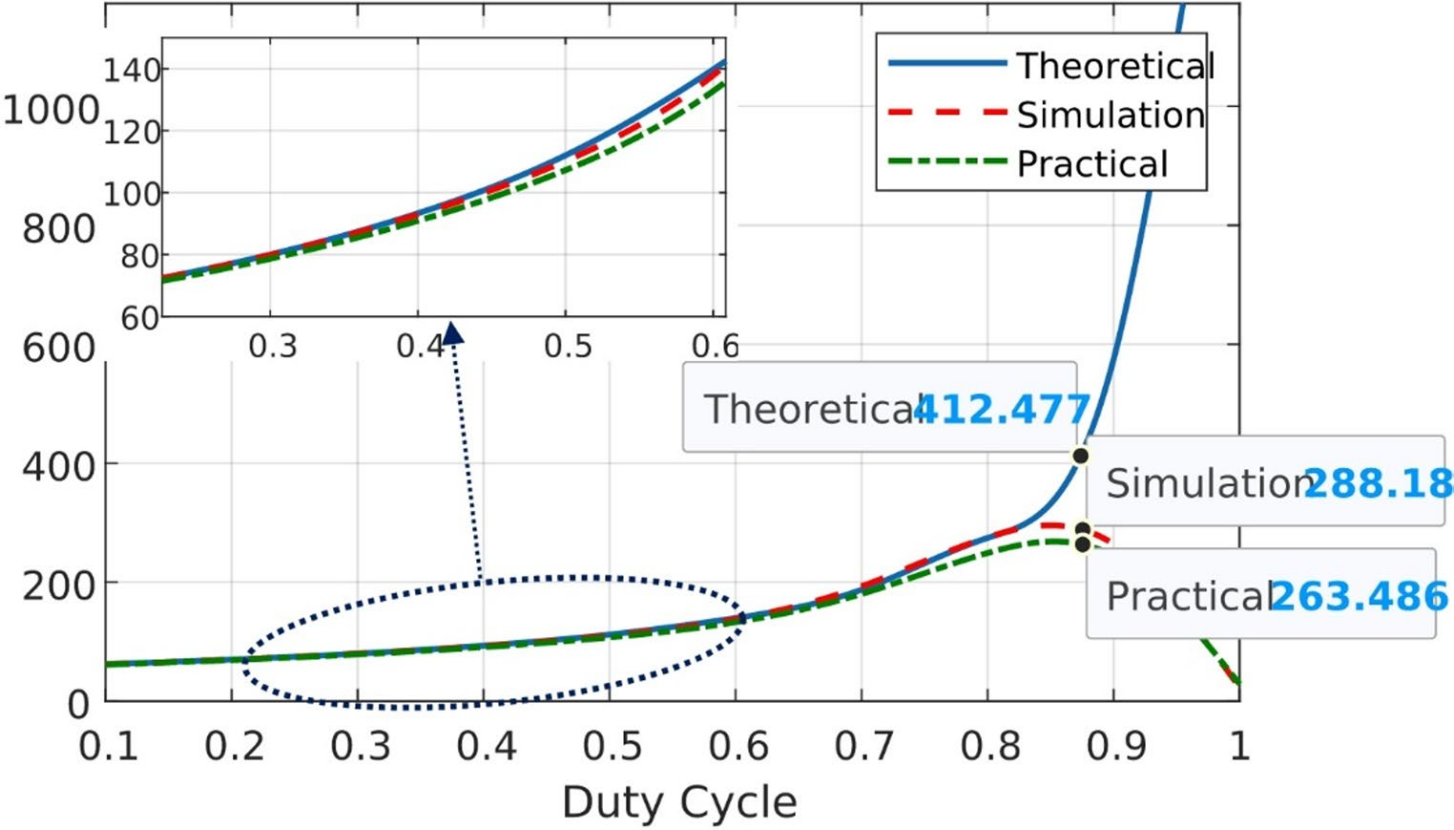
Output voltages vs. duty cycle of BBB-MPB in mode 4 (simulation and practical).

Figures 22 and 23 present the theoretical, simulation, and practical results of the voltage gain for modes 1, 2, and 3 at various duty ratios, respectively. Figure 24 present the theoretical, simulation, and practical results of the voltage gain for mode 4 at various duty ratios. In mode 4, as the duty cycle approaches extreme values, losses increase substantially, resulting in a reduced output voltage. This is illustrated in Fig. 24, where the output voltage decreases at a duty ratio higher than 80%.

Table 4 illustrates the inputs and outputs of BBB-MPB converter, where percentage error is calculated in accordance to theoretical and practical outputs. Furthermore, a comprehensive evaluation is performed to compare different MPB converters based on the number of switches (MOSFETs, IGBTs), inductors, capacitors, number of ports, and operation modes. The comparison is illustrated in.

Table 5.

**Table 4:** Theoretical, simulation and practical results for BBB converter in all four modes.

Input	Mode	Practical Output (V)	Simulation Output (V)	Theoretical Output (V)	Error (%)
PV Voltage 56.5 V	1	17.7	18.4 V	18.83 V	6
Grid Voltage 56.5 V	2	17.7	18.4 V	18.83 V	6
Battery Voltage 56.5 V	3	158.3	163.5 V	169.5 V	7
PV Voltage 56.5 V	4	102.83	113 V	114 V	9

**Table 5:** BBB-MPB converter comparison with prior converters.

MPB	*N*_t_	*N*_mi_	*N*_I_	*N*_C_	*N*_*P*_	Max. % Step-up	Max. % Step-down	Theoretical Gain Step-up	Theoretical Gain Step-down	Modes of operation
[19]	11	3	3	5	2	91.8%	91.8%	2D/1-D	D/2(1-D)	2
[20]	11	5	2	4	2	94.3%	94.4%	2/1-D	D/2	2
[21]	12	4	3	5	2	95%	95%	2/1-D	D/2	2
[22]	9	3	2	4	2	93.2%	94.1%	1 + D/1-D	D/2-D	2
[24]	9	4	2	3	2	97.6%	97.2%	D(D-2)/(1-D)^2^	D^2^/(1-D^2^)	2
[25]	12	8	2	2	3	90%	-	1/(1-D)	-	3
[26]	12	3	3	6	3	92.5%	92%	2/1-D	D/(1-D)	2
[27]	11	5	2	4	2	97%	96%	1 + D/(1-D)^2^	D^2^/(2-D)	2
Proposed BBB-MPB	13	7	3	3	3	94.3%	95%	1 + D/1-D, 1/1-D	D/2-D	4

where N_t_ is the total number of components, N_mi_ is the number of MOSFETs and IGBTs, N_I_ is the number of inductors, N_C_ is the number of capacitors, and N_P_ is the number of ports. As for the practical efficiency, Max. % is the maximum efficiency in either step up or step down. Furthermore, the converter in^25^ does not support step-down mode the only mode is step-up. Moreover, the MPB in^26^ supports only boost and buck-boost modes. In addition, the converter in^27^ has higher gain in step-up mode but with lower number of ports and lower number of working modes. As the proposed BBB-MPB has higher components than other converters, BBB-MPB also has a higher number of ports and more working modes than most other MPB converters.

**Fig. 25:**
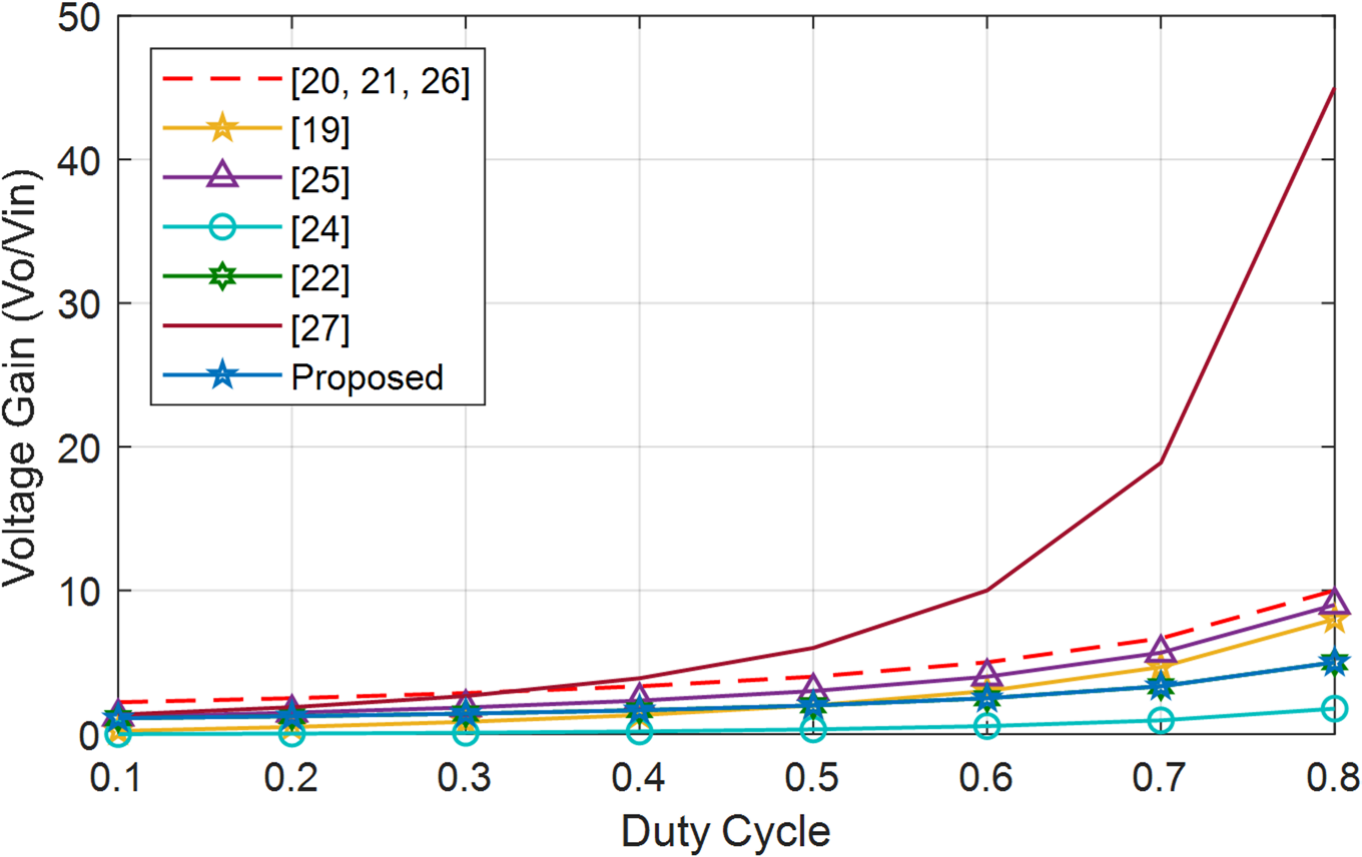
Converters comparison in step-up mode.

**Fig. 26:**
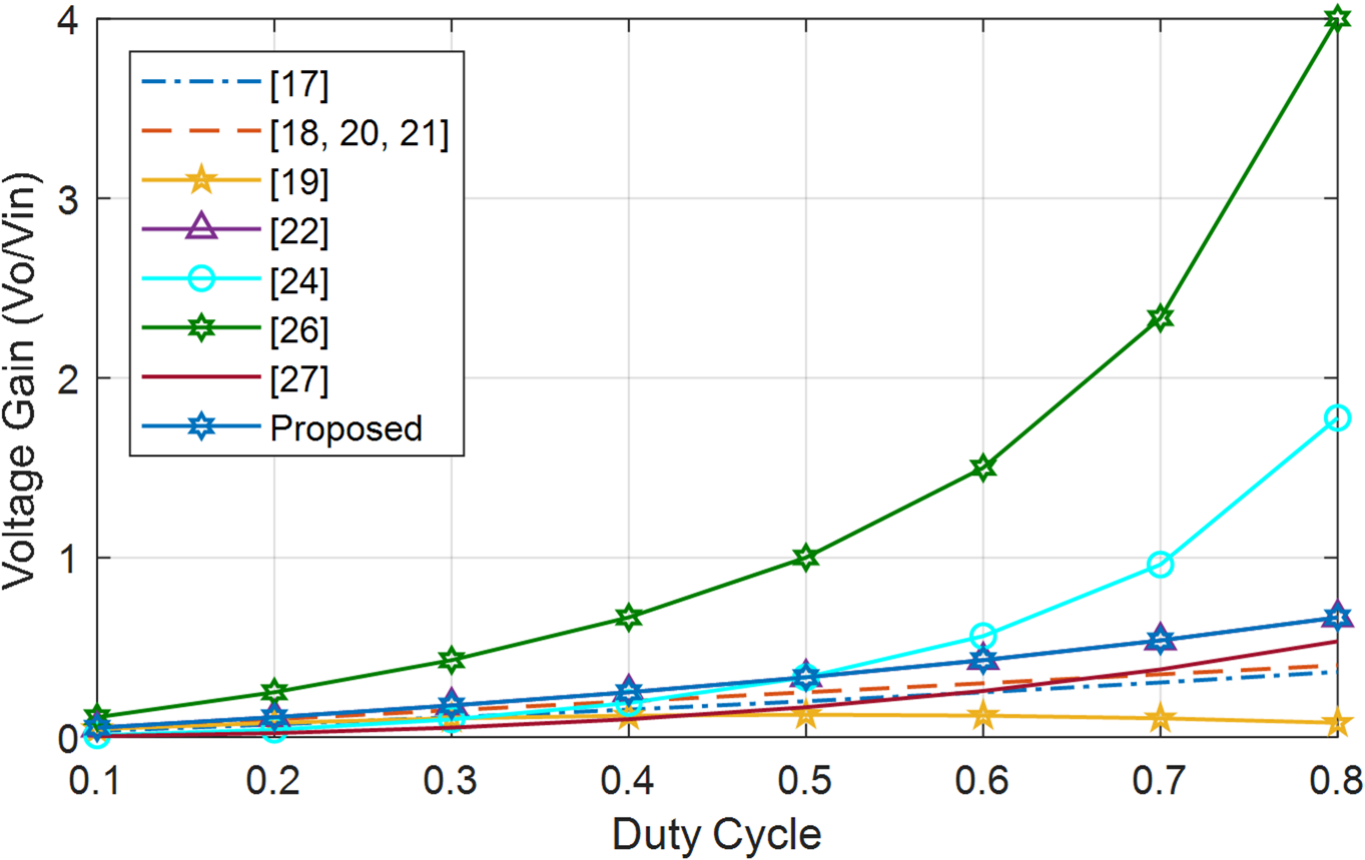
Converters comparison in step-down mode.

Figures 25 and 26 represent voltage gain comparison of all converters for step-up and step-down modes. Compared to other BDCs and MPBs, BBB-MPB has a higher voltage gain than^24^ and same as^22^ but lower than the rest of converters in step-up mode. BBB offers additional operational modes and features 3 ports, as opposed to the 2 ports found in all converters. BBB-MPB almost has a linear gain in step-down mode. The gain is equal to that in^22^.

In summary, the results indicate that BBB exhibits higher gain compared to other converter in step-up mode, despite it is still lower than that of numerous other converters. Furthermore, this MPB converter has four distinct modes of operation (PV2V, G2V, V2G, and PV2G), which are not available in any other converter. Moreover, BBB and BBS exhibit higher efficiency as compared to other converters.

### Efficiency and power loss analysis of BBB-MPB

**Fig. 27:**
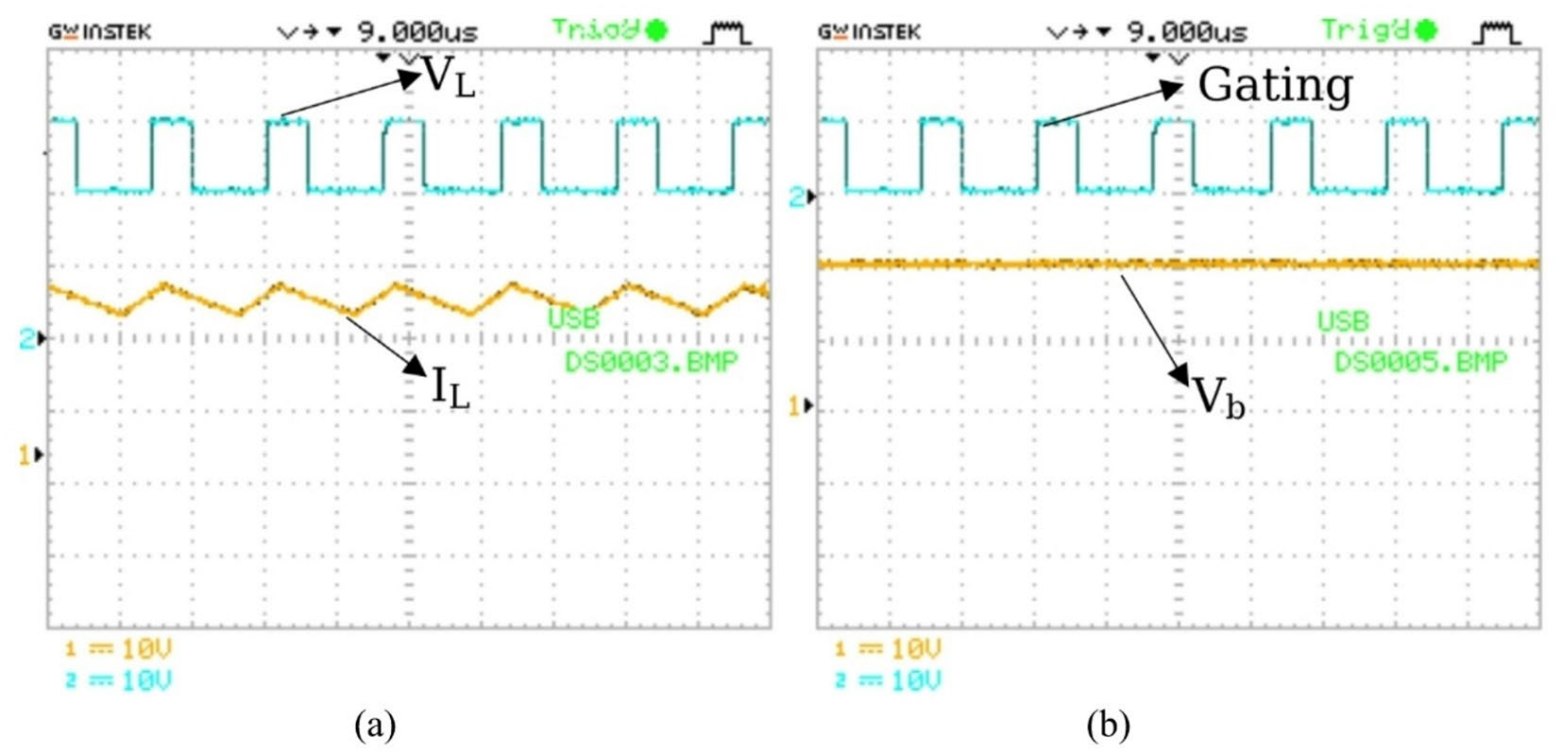
Experimental voltage and current waveforms for BBB-MPB at 30% duty ratio in modes 1 and 2 (a) inductor voltage and current waveforms (I_L_= 15 A); (b) output voltage and gating signal.

**Fig. 28:**
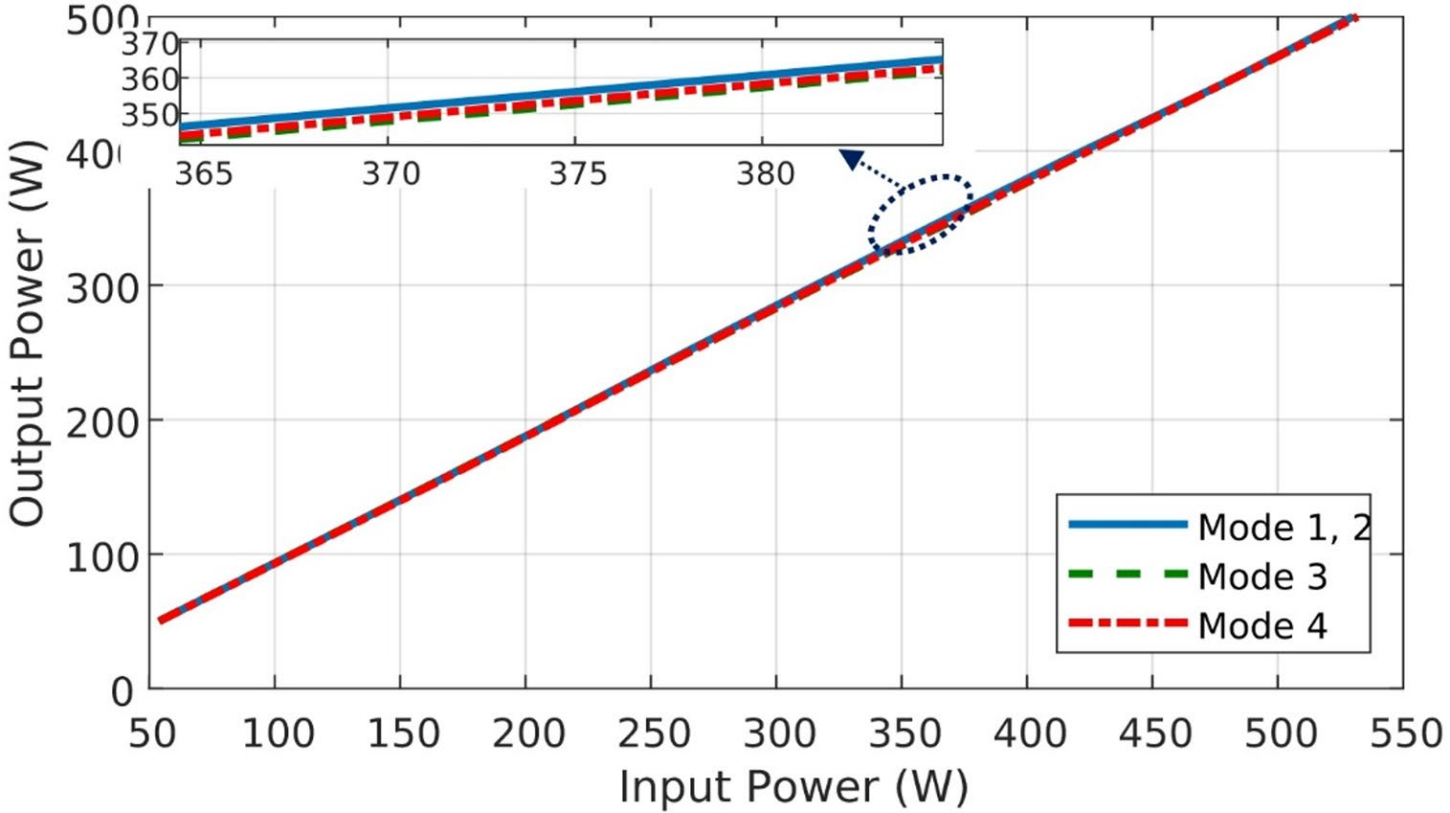
Input power vs. output power of BBB-MPB.

**Fig. 29:**
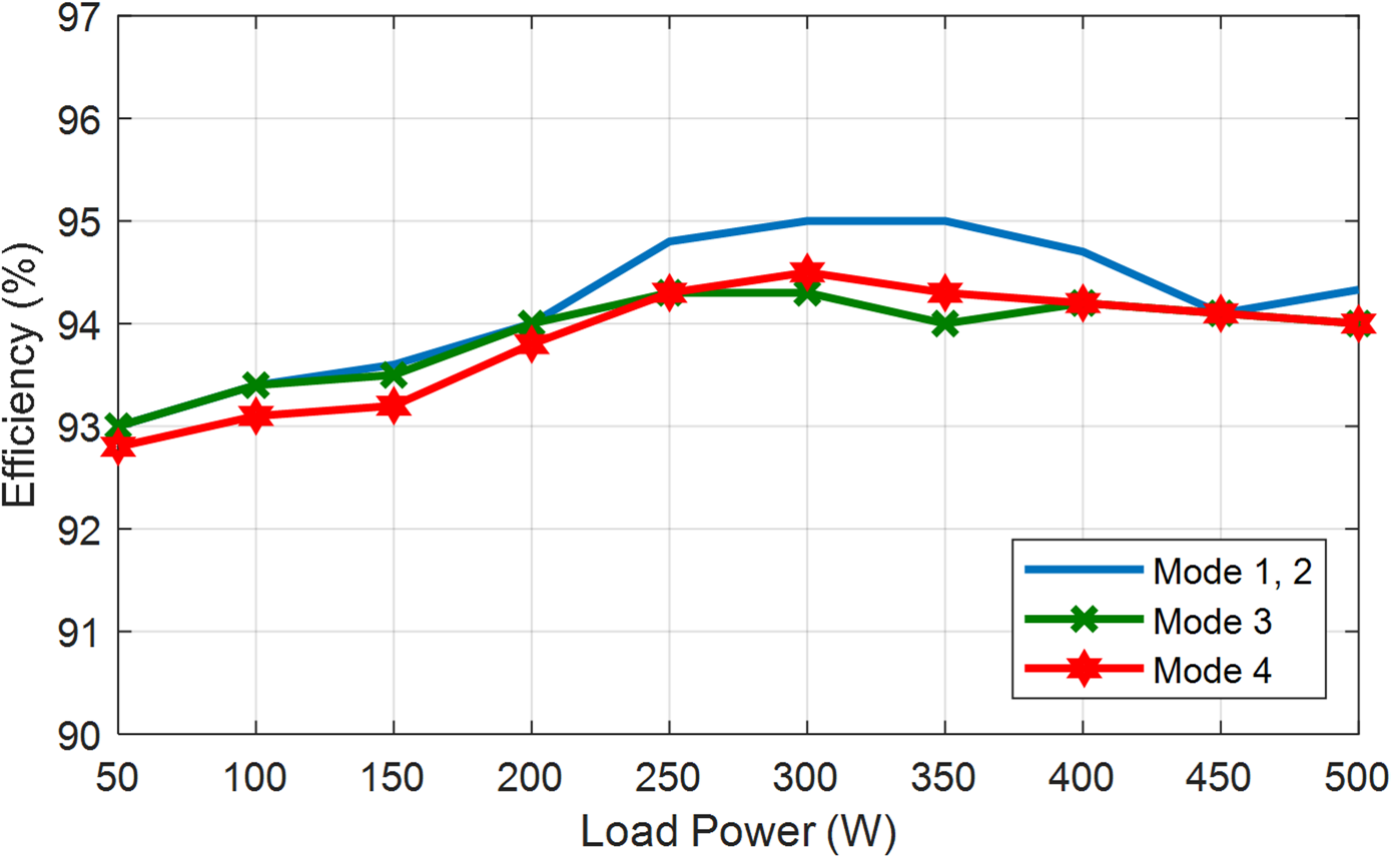
Efficiency vs. load power for BBB-MPB.

For more clarification Fig. 27 presents the inductor (L_1_) current and voltage for modes 1 and 2. In addition to the output voltage and gating signal at a duty ratio of 30% for modes 1 and 2. BBB-MPB exhibits the peak efficiency of modes 1 and 2 at the duty ratio of 30% and a power of 300 W. Figure 27 (a) represents the inductor voltage and current waveforms. The inductor current is measured to be 15 A, and the voltage is 19.8 V. In addition, Fig. 27 (b) shows the output voltage and the gating signal. The input current is measured to be 2.788 A at a voltage (V_pv_) of 113 V. Figure 28 shows the input and output power of the BBB-MPB in all four modes. The experimental efficiency of the proposed BBB-MPB for all four modes is illustrated in Fig. 29 at various load powers. Figure 29 shows that the peak efficiency for modes 1 and 2 is 95%. The peak efficiency for mode 3 is 94.3%, and for mode 4, is 94.5%. The temperature of the power electronic device’s heat sink is measured to be 30^o^ C. As the temperature rises, the on resistance increases and so power dissipation. While the drain current decreases as temperature increases.

The conduction loss of each switch is expressed as:

43 $$\:{P}_{cond.}={i}_{S}^{2}\text{*}{r}_{S}$$

The switching loss is formulated as:

44 $$\:{P}_{switching}=\frac{{t}_{on}+{t}_{off}}{2}\text{*}{F}_{S}\text{*}{V}_{S}\text{*}{i}_{SW}$$

Finally, the inductor loss is expressed as :

45 $$\:{P}_{inductor}=Core\:loss+Copper\:loss{=i}_{L}^{2}\text{*}{r}_{L}+2k\text{*}{{F}_{S}}^{x}\text{*}{B}^{y}\text{*}{V}_{C}$$

Where i_SW_ is the switch current, t_on_ and t_off_ are the on and off time respectively, i_L_ is the inductor current, k is the core material constant, B is the peak flux density, x is the frequency exponent, y is the flux density exponent, and V_C_ is the effective core volume. The loss distribution of the BBB-MPB at 300 W load power at a duty ratio of 30% for modes 1, 3, and 4 is measured as 15.78 W, 17.46 W, and 18.8 W, respectively. Table 6 shows the detailed losses in each mode. Table 6 illustrates the losses due to conduction, switching of the switches, and inductors for each mode.

As for Efficiency Sustainability, Projected lifespan: is longer than 50,000 h (6 years) at 20 kHz/25°C ambient, based on:

- Arrhenius model: 10 °C rise halves component lifespan.
- Electrolytic capacitor aging: 2% efficiency drop after 10,000 h (estimated via Lelon datasheet).
- De-rating: Components operate at 70–80% of max ratings to ensure longevity.

**Table 6:** Experimental S.

Loss distribution	Mode 1 and 2	Mode 3	Mode 4
Switching losses	3.94 W	4.43 W	4.83 W
Conduction losses	8.67 W	8.73 W	9.44 W
Inductor losses	3.16 W	4.3 W	4.5 W
Total losses	15.78 W	17.46 W	18.77 W

In the prototype, GT35J321 and IRGB15B60KD IGBTs are employed with a switching frequency of 20 kHz, ensuring operation within the recommended range to suppress tail-current effects. Both simulation and experimental results confirm the absence of tail-current issues. The converter achieves peak efficiencies of 94–95%, with device temperatures maintained around 30 °C, indicating acceptable switching losses and reliable operation. Further efficiency enhancement is expected by adopting wide bandgap devices (SiC/GaN).

### Comparison with existing products

The BBB converter (as applied to EV DC fast charging) is compared with commercial off-the-shelf (COTS) charging systems in terms of performance, cost, and applicability^28^. The comparison is shown in Table 7.

**Table 7:** Comparison of BBB vs. off-the-shelf products.

Metric	BBB	COTS (ABB, Tesla, etc.)
Power Range	Scalable (multi-port)	Fixed (50 kW, 150 kW)
Efficiency	90–93%	93–96% (optimized)
Voltage Range	Wide (200–1000 V, adjustable gain)	Fixed (400–800 V)
Component Cost	High (discrete design)	Low (integrated modules)
Renewable Integration	Built-in PV/grid support	Requires external systems

Unique Advantages of the BBB Converter in EV Charging:

- Multi-Port Flexibility:

1. Integrates PV, battery storage, and grid in a single unit (e.g., solar-powered charging stations).
2. COTS chargers typically require external battery buffers.

- Dynamic Voltage Adaptation:

1. Adjusts output voltage (200–1000 V) for legacy and 800 V EVs without additional DC-DC stages.
2. Commercial chargers often need separate hardware for 400 V/800V compatibility.

- Renewable Energy Optimization:

1. Modes 1–4 enable direct PV2V or G2V charging.

However, BBB has some limitations in comparison to the commercial DC chargers.

- Efficiency:

1. Commercial chargers use SiC/GaN-based modules (e.g., Wolfspeed, STMicroelectronics) for 96%+ efficiency.
2. BBB’s discrete design may lag by 2–3%.

- Cost:

1. COTS: Low at scale (integrated design).
2. BBB: High (discrete components for prototype).

The prototype is designed with voltage ratings of 56.5 V at the input and 17.7–158.3 V at the outputs depending on the mode of operation, constrained by the available laboratory power supplies and the safe operating limits of the selected MOSFETs and IGBTs. These ratings enabled experimental validation of the proposed topology under safe conditions without risking device failure. As summarized in Table 7.

The prototype voltages are lower than those typically employed in commercial EV chargers (400–800 V). Nevertheless, the proposed converter is inherently scalable to higher voltage levels through the use of appropriately rated semiconductor devices. Hence, the present low-voltage prototype serves as a proof of concept, while practical implementation at standard EV charging levels is straightforward.

## System dynamics

### Small-signal analysis

This section inspects the small-signal characteristic of the presented topology. Small-signal model is usually described by state-space equations. The current of the two inductors and the voltage of the capacitors are the state space variables. The small-signal equations are derived by separating first-order variables and removing second-order parameters. The above procedure is applied to the converter. It is worth noting that parasitic elements of components have not been considered in the model due to their small values and due to obtaining a simple transfer function^29,30^.

For modes 1, 2 the same transfer function is obtained as it is the same construction.

Perturb the duty cycle and other variables:

46 $$\:d\left(t\right)=D+\widehat{d},\:V\left(t\right)=V+\widehat{v},\:{I}_{L}\left(t\right)={I}_{L}+\widehat{{i}_{L}},\:{V}_{o}\left(t\right)={V}_{o}+\widehat{{v}_{o}}$$

Small-Signal Model for Mode 1 (CCM)

47 $$\:\left(D+\widehat{d}\right)\left(\frac{{V}_{pv}+{\widehat{v}}_{pv}-{V}_{b}-{\widehat{v}}_{b}}{2}\right)+\left(1-D-\widehat{d}\right)\left(-{V}_{b}-{\widehat{v}}_{b}\right)=0$$

Substitute steady state V_b_

$$\:{V}_{b}=\frac{D}{2-D}{V}_{pv}\:$$

48 $$\:\frac{D}{2}{\widehat{v}}_{pv}-\frac{D}{2}{\widehat{v}}_{b}+\left(\frac{{V}_{pv}+\left(2-2D\right)}{2\left(2-D\right)}\right)\widehat{d}-(1-D){\widehat{v}}_{b}=0$$

Simplify

49 $$\:{\widehat{v}}_{b}\left(s\right)\left(\frac{2-D}{2}\right)=\frac{{V}_{pv}\left(1-D\right)}{2-D}\widehat{d}\left(s\right)$$

The static control to output transfer function is obtained as

50 $$\:\frac{{\widehat{v}}_{b}\left(s\right)}{\widehat{d}\left(s\right)}=\frac{2{V}_{pv}(1-D)}{{(2-D)}^{2}}$$

Extend the same equation but with dynamics

51 $$\:L\frac{d\widehat{{i}_{L}}}{dt}=\frac{D}{2}{\widehat{v}}_{pv}+\frac{{V}_{pv}}{2}\widehat{d}-\left(1-D\right){\widehat{v}}_{b}+{V}_{b}\widehat{d}$$

52 $$\:C\frac{d\widehat{{V}_{b}}}{dt}=\left(1-D\right){\widehat{i}}_{L}-\frac{{\widehat{v}}_{b}}{R}+{I}_{L}\widehat{d}$$

Where $$\:{I}_{L}=\frac{{V}_{b}}{R(1-D)}$$.

The final dynamic transfer function is obtained as follows

53 $$\:{G}_{1,\:2}\left(s\right)=\frac{{\widehat{v}}_{b}\left(s\right)}{\widehat{d}\left(s\right)}=\frac{2{V}_{pv}(1-D)(1-s\frac{L}{R{(1-D)}^{2}})}{{\left(2-D\right)}^{2}(1+s\frac{L}{R{\left(1-D\right)}^{2}}+{s}^{2}\frac{LC}{R{\left(1-D\right)}^{2}})}$$

By substituting with numerical values used in previous section the transfer function in its numerical form is obtained.

54 $$\:{G}_{1,\:2}\left(s\right)=\frac{{\widehat{v}}_{b}\left(s\right)}{\widehat{d}\left(s\right)}=\frac{25.11(1-s\frac{1}{25000})}{(1+s\frac{1}{17600}+{s}^{2}\frac{1}{1.25\times\:{10}^{8}})}$$

Same analysis is done for modes 3, and 4 as the configuration changes and the transfer function is obtained as:

55 $$\:{G}_{3}\left(s\right)=\frac{{V}_{g}\left(s\right)}{D\left(s\right)}=\frac{452(1-\frac{s}{66667})}{\left(1+\frac{s}{17600}+\frac{{s}^{2}}{1.25\times\:{10}^{8}}\right)}$$

56 $$\:{G}_{4}\left(s\right)=\frac{{V}_{g}\left(s\right)}{D\left(s\right)}=\frac{226(1-\frac{s}{25000})}{\left(1+\frac{s}{17600}+\frac{{s}^{2}}{1.25\times\:{10}^{8}}\right)}$$

In order to add parasitic elements to the transfer function the functions are obtained as follows

57 $$\:{G}_{1,\:2}\left(s\right)=\frac{{\widehat{v}}_{b}\left(s\right)}{\widehat{d}\left(s\right)}=\frac{2{V}_{pv}(1-D)(1-s\frac{L(1-D)}{{R}_{eq1}(2-D)})}{{\left(2-D\right)}^{2}(1+s\frac{L+C({R}_{eq1}{r}_{C}+{R}_{eq1}{r}_{L})}{{R}_{eq1</span>}\left(2-D\right)}+{s}^{2}\frac{LC}{R{\left(2-D\right)}^{2}})}$$

58 $$\:{G}_{3}\left(s\right)=\frac{{\widehat{v}}_{g}\left(s\right)}{\widehat{d}\left(s\right)}=\frac{{V}_{b}(1+D)(1-s\frac{L(1+D)}{{2R}_{eq3}(1-D)})}{{\left(1-D\right)}^{2}(1+s\frac{L+C({R}_{eq3(}{r}_{C}+{r}_{L})}{{R}_{eq3}}+{s}^{2}LC)}$$

59 $$\:{G}_{4}\left(s\right)=\frac{{\widehat{v}}_{g}\left(s\right)}{\widehat{d}\left(s\right)}=\frac{{V}_{pv}(1-s\frac{L}{{R}_{eq4}(1-D)})}{{\left(1-D\right)}^{2}(1+s\frac{L+C({R}_{eq4(}{r}_{C}+{r}_{L})}{{R}_{eq4}}+{s}^{2}LC)}$$

Where

60 $$\:{R}_{eq1}=R\left|\right|\frac{({r}_{L}+{r}_{s}+{r}_{d})}{1-D}$$

61 $$\:{R}_{eq3}=R\left|\right|\frac{({r}_{L}+{r}_{s}+{r}_{d})}{D}$$

62 $$\:{R}_{eq4}=R+{r}_{T1}+{r}_{T2}$$

**Fig. 30:**
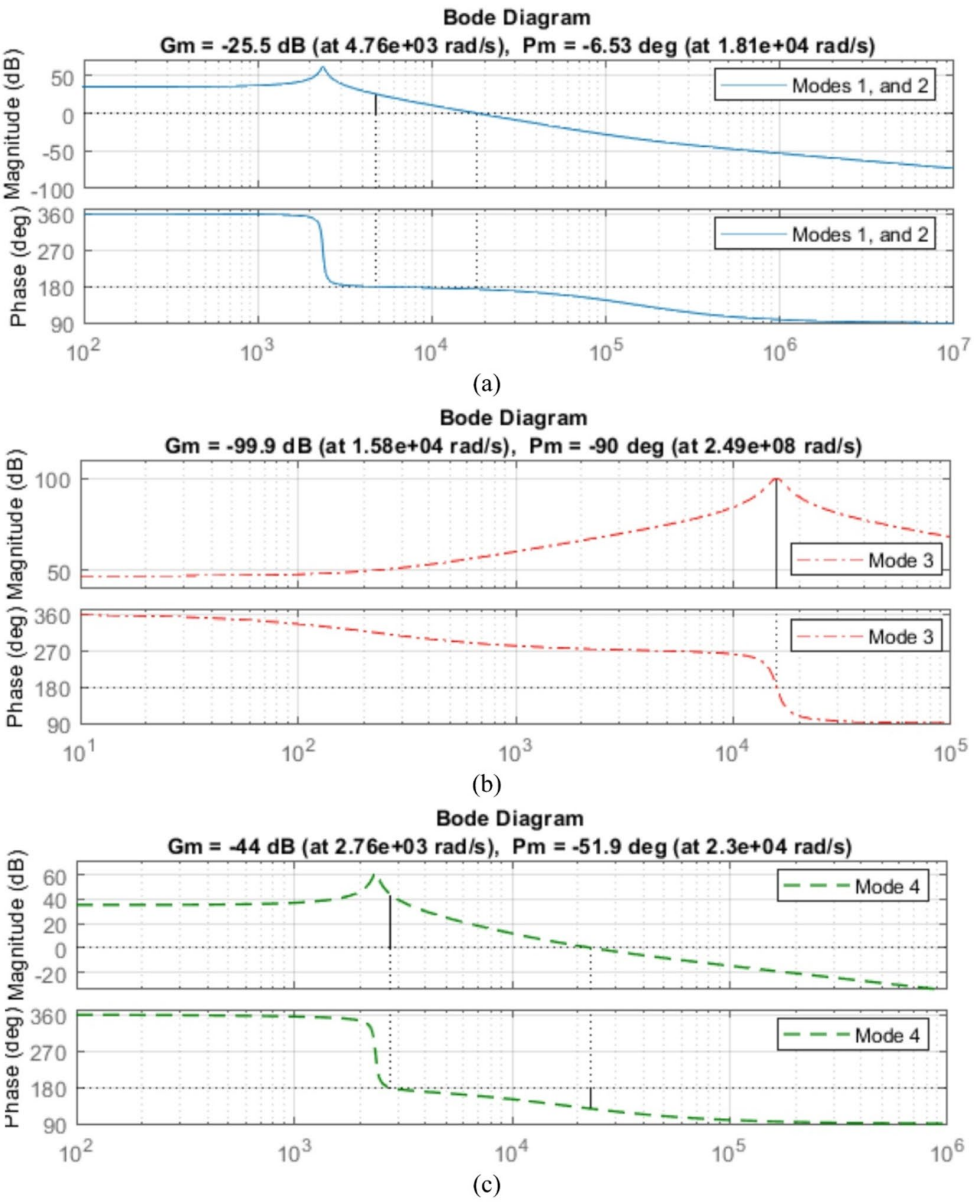
Bode Plot of the Four Modes (**a**) Modes 1, and 2; (**b**) Mode 3; (**c**) Mode 4.

The bode plots for the proposed converter is shown in Fig. 30.

**Fig. 31:**
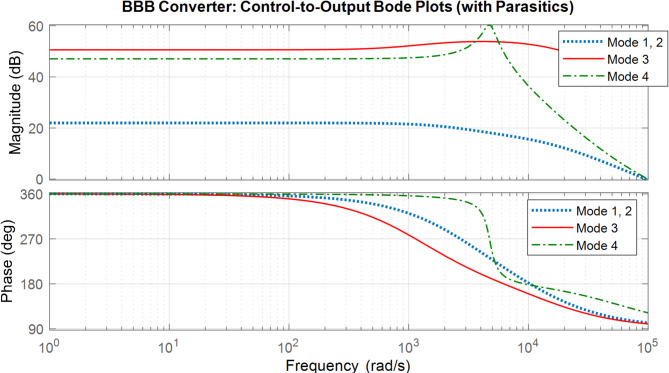
Bode Plot of the Four Modes (**a**) Modes 1, and 2; (**b**) Mode 3; (**c**) Mode 4 with non ideal elements.

As for the non-ideal bode plot for the 4 modes. Figure 31 show the 4 modes bode plot. Bode plot analysis revealed that none of the modes exhibit inherent closed-loop stability under uncompensated conditions. Specifically, all four modes demonstrated negative gain margins and inadequate phase margins at the designated operating frequency of 20 kHz, indicating a high risk of instability or oscillatory. By applying appropriate compensation and control strategies, the BBB converter can be reliably stabilized across all modes, making it a viable candidate for multi-port energy management applications such as photovoltaic charging, vehicle-to-grid (V2G), and grid-assisted operations.

### Dynamic performance

**Fig. 32:**
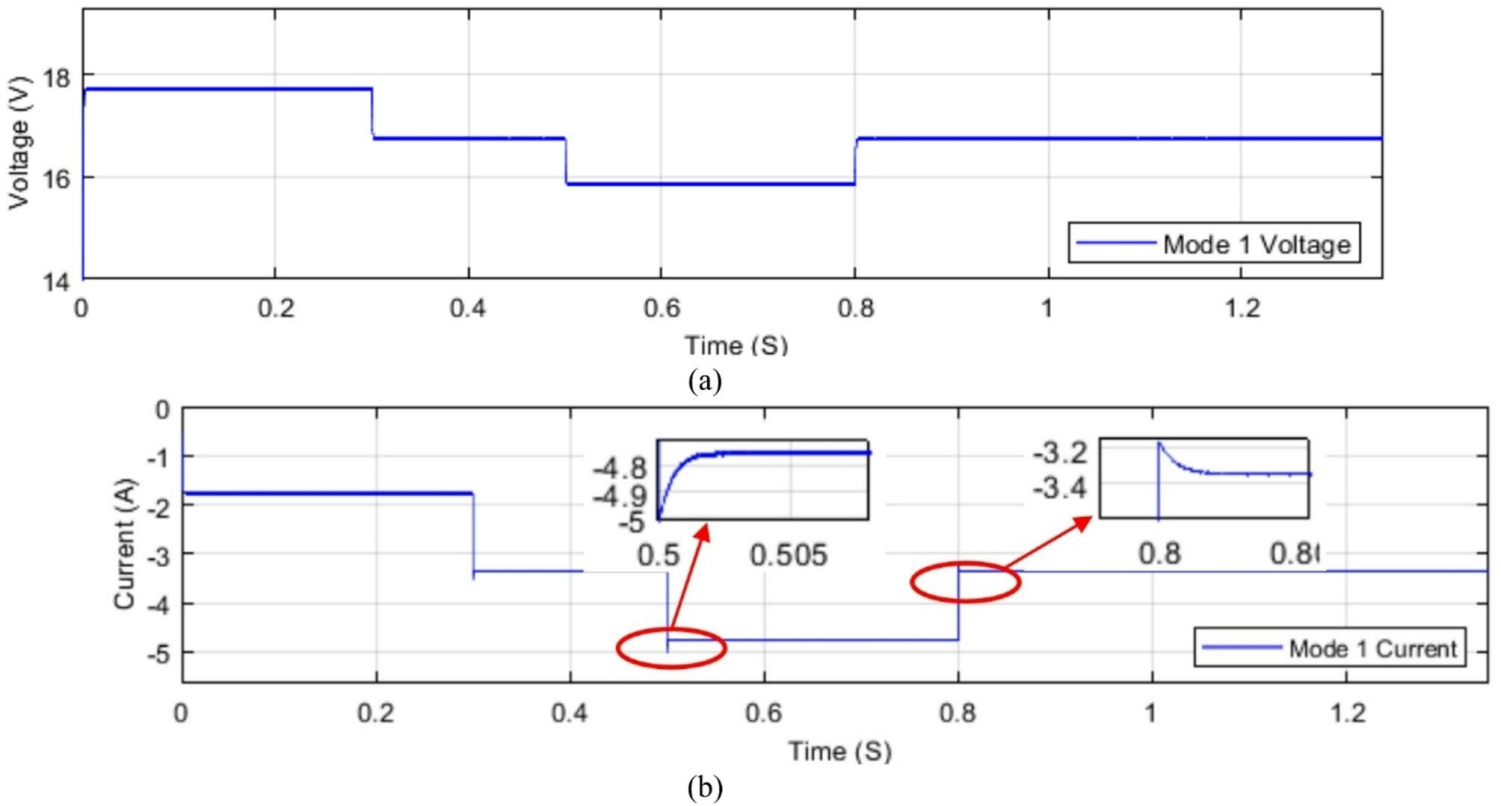
Dynamic change in load conditions of modes 1, and 2 (**a**) voltage dynamic performance; (**b**) current dynamic performance.

**Fig. 33:**
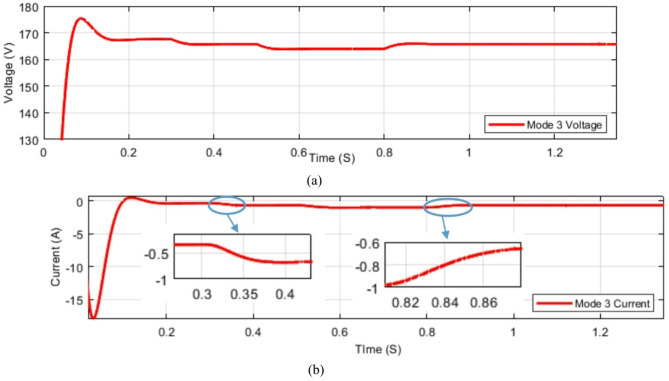
Dynamic Change in load conditions of mode 3 (**a**) voltage dynamic performance; (**b**) current dynamic performance.

The converter is tested under dynamic output. While the output changes in a step change the voltage and current are tested in simulations. Test is done for modes 1, 2, and 3 and the results are shown in Figs. 32 and 33. The dynamic change in load is done at 0.3 S, 0.5 S, and 0.8 S as observed in below figures.

As observed from the above figures the system is dynamically stable with the sudden change in load conditions. However a controller is recommended as stated before in this section.

## Conclusion

This research offered two new MPB converters for the integration of renewable energy in off-board charging stations of EVs. The proposed BBB and BBS converters are studied using theoretical approach. In this paper, only CCM is studied for the proposed converters, as it will be used as an off-board chargers for EVs. BBB and BBS converters have four modes of operation, which is the novelty of these MPBs. Both converters can work under four modes of operation (PV2V, G2V, V2G, and PV2G). The theoretical model is studied for all four modes and for the selection of components to insure a CCM operation. BCM and DCM theoretical studies are also obtained. Voltage and current stresses are also expressed for an overall theoretical study. The following conclusion can be stated:

1. Theoretical analysis is verified using simulations by MATLAB/SIMULINK.
2. A thorough comparative study is carried out using recent comparable MPBs. This study confirmed that the BBB-MPB could achieve high voltage gain and high efficiency (94.5%).
3. With a lower number of working modes, some recent MPBs have slightly higher efficiency and gain.
4. A prototype of the BBB-MPB is implemented and tested. Experimental results are also used to validate the operating and performance of the converter.
5. Comparison with off-the-shelf products is done and show that BBB converter is not a drop-in replacement for commercial EV chargers but excels in niches requiring flexibility and renewable integration and needs some improvements to be a replacement.
6. BBB converter require a compensation control system as it has marginal stability according to the performance study.
7. Future work will focus on adding advanced control and MPPT algorithms to improve stability, dynamic response, and power output.
8. Soft-switching techniques will be explored to reduce losses and improve efficiency.

## Data Availability

The datasets generated during and/or analysed during the current study are available from the corresponding author on reasonable request.
